# Spontaneous NETosis in diabetes: A role of hyperglycemia mediated ROS and autophagy

**DOI:** 10.3389/fmed.2023.1076690

**Published:** 2023-02-20

**Authors:** Anam Farhan, Ghulam Hassan, Sheikha Hina Liaqat Ali, Zainab Yousaf, Kandeel Shafique, Amir Faisal, Bilal bin Younis, Shaper Mirza

**Affiliations:** ^1^Department of Life Sciences, Syed Babar Ali School of Science and Engineering, Lahore University of Management Sciences, Lahore, Pakistan; ^2^Sakina Institute of Diabetes and Endocrinology Research (SiDER), Shalamar Hospital, Lahore, Pakistan

**Keywords:** type 2-diabetes, reactive oxygen species, NETosis, phagocytosis, autophagy, NADPH oxidase

## Abstract

Type 2-diabetes, particularly poorly controlled diabetes, is a risk factor for several infections such as lower respiratory tract and skin infections. Hyperglycemia, a characteristic downstream effect of poorly controlled diabetes, has been shown to impair the function of immune cells, in particular neutrophils. Several studies have demonstrated that hyperglycemia-mediated priming of NADPH oxidase results in subsequent elevated levels of reactive oxygen species (ROS). In healthy neutrophils, ROS plays an important role in pathogen killing by phagocytosis and by induction of Neutrophil Extracellular Traps (NETs). Given the key role of ROS in autophagy, phagocytosis and NETosis, the relationship between these pathways and the role of diabetes in the modulation of these pathways has not been explored previously. Therefore, our study aimed to understand the relationship between autophagy, phagocytosis and NETosis in diabetes. We hypothesized that hyperglycemia-associated oxidative stress alters the balance between phagocytosis and NETosis by modulating autophagy. Using whole blood samples from individuals with and without type 2-diabetes (in the presence and absence of hyperglycemia), we demonstrated that (i) hyperglycemia results in elevated levels of ROS in neutrophils from those with diabetes, (ii) elevated levels of ROS increase LCIII (a marker for autophagy) and downstream NETosis. (iii) Diabetes was also found to be associated with low levels of phagocytosis and phagocytic killing of *S. pneumoniae*. (iv) Blocking either NADPH oxidase or cellular pathways upstream of autophagy led to a significant reduction in NETosis. This study is the first to demonstrate the role of ROS in altering NETosis and phagocytosis by modulating autophagy in type 2-diabetes.

**GRAPHICAL ABSTRACT:**
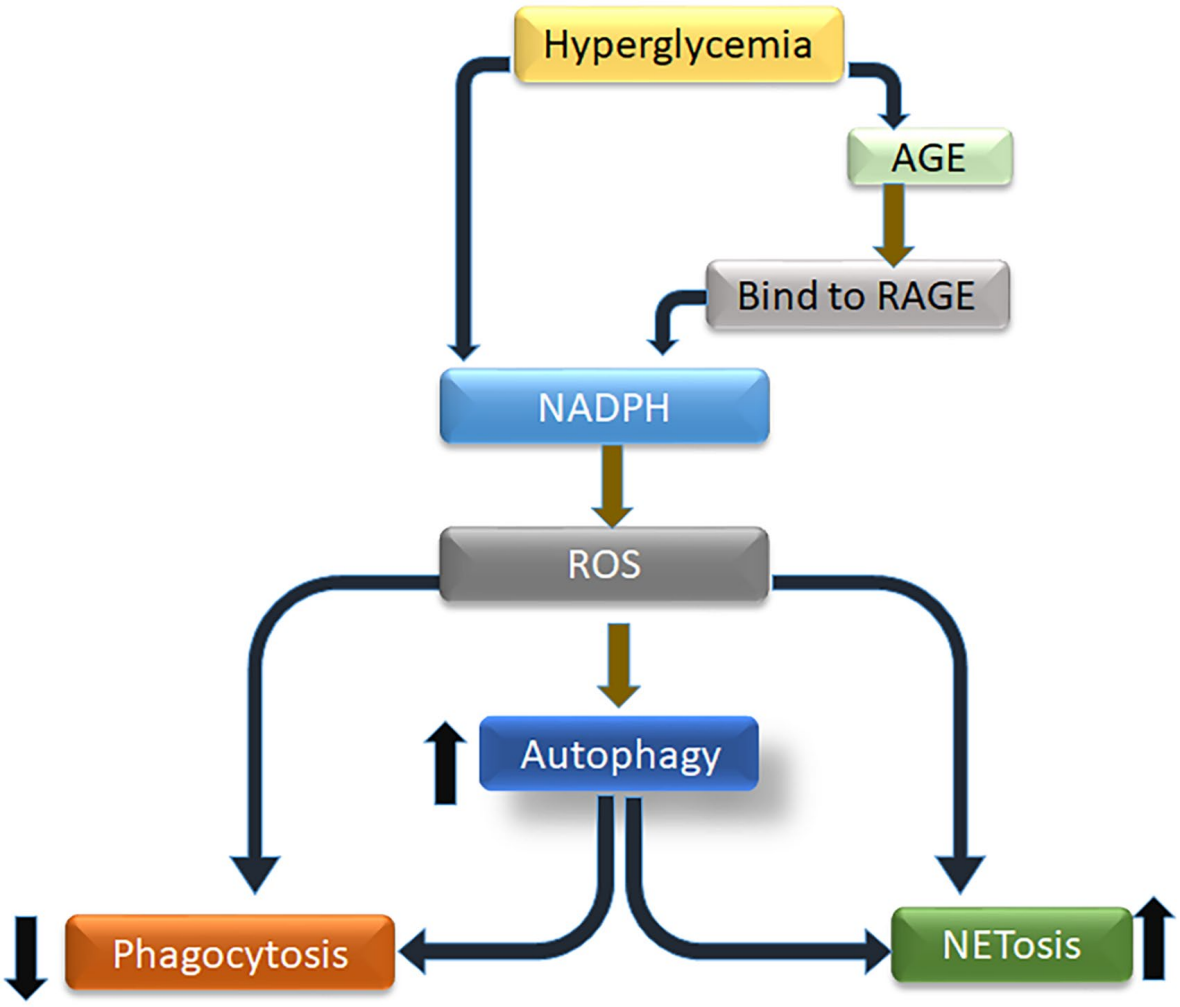

## 1. Introduction

Type 2-diabetes mellitus (T2D), a metabolic syndrome characterized by chronic hyperglycemia, remains one of the largest emerging threats to global health in the 21st century. During the last decade, evidence has emerged linking T2D to an increased risk of several infections (1–3), of which the most important are respiratory tract infections (4, 5) including tuberculosis (TB), pneumonia and influenza (6, 7). In addition to respiratory tract infections, diabetes is also associated with a 1.5-fold increased risk of surgical site infections (8), a 2-fold increased risk of urinary tract infections (UTIs) (9), and a 2-to 3-fold increased risk of bacteremia (10). This increased susceptibility is attributed to impairments in sentinel cells of the immune system, such as neutrophils (11).

Neutrophils (PMNs), one of the principal effector cells of the immune system, are short-lived phagocytes (12). They are known for their role in the surveillance and killing of invading microbes. By adopting several strategies, these leukocytes provide defense against a broad range of pathogens, thus limiting their spread (13). Recently, new horizons of neutrophil activity have been revealed, suggesting their long-term involvement in infection and inflammation (14, 15). Brinkmann and co-workers identified a novel strategy of trapping and killing microbes by neutrophils and coined the term neutrophil extracellular trap (NET) (16). While phagocytosis requires engulfing and killing extracellular bacteria, in NETosis, neutrophils undergo multiple morphological changes to release NETs that are characterized by a web of DNA decorated with cytoplasmic antimicrobial compounds (17–19).

While the molecular mechanisms driving NETosis remain largely unknown, the role of NADPH oxidase-dependent ROS in the induction of NETosis is well established (20–22). The NADPH oxidase is a multi-subunit enzyme complex, with subunits localized in both cytoplasm and cell membrane. Upon activation, cytosolic subunits migrate to the membrane resulting in the formation of an active enzyme complex (23). Reactive oxygen species thus generated are considered an important second messenger required for NETs generation and several other processes, including phagocytosis and autophagy (24, 25). Autophagy is a well-conserved cellular mechanism that, under certain conditions, allows for the degradation of cytoplasmic material and organelles to recycle their constituents. It also plays a significant role in infection and inflammation, eliminating phagocytosed microbes and limiting inflammasome activation (26, 27). Autophagy-related signaling has been implicated in NETosis (28, 29).

NETosis is a physiological process that, although exacerbated in diabetes, is instrumental to protecting against invasive infections (22). However, exacerbation of NETs in T2D is far from being completely understood. It has recently been described that high glucose *in vitro* and hyperglycemia *in vivo* increase the release of NETs, characterized by elevated levels of circulating peptidyl-arginine-deiminase, an enzyme important in chromatin decondensation and DNA release, in T2D (30–34). However, these studies did not address the biological processes associated with the observed increase in NETosis, neither did these studies elaborate on associations between hyperglycemia, NETosis, phagocytosis and autophagy. It is plausible that, NETosis and autophagy operates as partners for carrying out antibacterial activity in a coordinated/cooperative manner.

While NETs formation is intended to have protective effect against pathogen, it can be detrimental to the surrounding tissues (35–37). NETs have been shown to cause both epithelial and endothelial cytotoxicity to respiratory cells during pneumonia (38, 39). It is therefore likely that high bacterial burden and toxicity from protective mechanisms like NETosis may lead to poor outcomes of pneumonia in those with diabetes. It therefore makes it imperative to understand these mechanisms in diabetes and identify relevant molecules and pathways that can be harnessed to reduce the burden of infection in those with diabetes.

The current study is therefore designed to expand our knowledge regarding the molecular cross-talk among pathways that regulate neutrophil antibacterial activity. This understanding will likely facilitate manipulation of the neutrophil machinery in chronic diseases such as diabetes for successful design of future inflammation modulatory therapeutics.

## 2. Materials and methods

The human subject’s research has been approved by the Lahore University of Management Sciences (LUMS) and by Shalamar Hospital Institutional Review Boards (IRB) to ensure risks to humans are minimized. Written informed consent were obtained from all the participants, and appropriately documented according to all rules and regulations for compliance with the University IRB.

### 2.1. Sample size

Primary goal of this study is to determine the role of hyperglycemia mediated oxidative stress on activation of autophagy and subsequent NETosis in individuals with and without type 2-diabetes. Using convenient sampling, blood samples were collected from a total of 60 participants, which included 30 individuals with diagnosed T2D and 30 age and sex matched healthy control.

#### 2.1.1. Inclusion criteria

Individuals 18 years and older diagnosed with type 2-diabetes were included in the study. Diabetes among study participants was either self-reported or doctor diagnosed. For diagnosis, diabetes was defined as follows: A fasting blood glucose of >100 mg/dl, glycated hemoglobin (HbA1c) of >6.5% and on medication for glucose (ADA guidelines 2010). Poorly controlled diabetes was further defined as fasting blood glucose of >126 mg/dl and HbA1c of >8% and on medication for glucose. Healthy control were defined as; age and sex matched individuals with no diagnosis of diabetes HbA1c <5.6%, fasting blood glucose of <100 mg/dl and not on medication for diabetes.

#### 2.1.2. Exclusion criteria

Participants were excluded from the study if they confirmed to any of the following (i) Individuals on antibiotics for any current infections (ii) diagnosed with tuberculosis and on ATT and (iii) HIV positive individuals.

### 2.2. Recruitment and informed consent

Participants who confirmed to our inclusion and exclusion criteria were approached and were requested to participate in the study. Participants with type 2-diabetes were recruited from Sakina Institute of Diabetes and Endocrinology Research (SiDER) at Shalamar Hospital, a 250 bed private tertiary care hospital in the south of the city. Whereas, age and sex matched healthy controls were recruited from either SiDER or from outpatient clinic at the department of medicine, Shalamar Hospital. Participants presenting at SiDER diabetes clinic or outpatient clinic in the department of medicine were invited to participate. Study was explained to each participant in local language that they were most familiar with, which include either Urdu (national language) or Punjabi (language of the natives of province of Punjab). Individuals who agreed to participate in the study were given a consent form in the language that they were most comfortable with (English, Urdu or Punjabi). Following signing of consent forms, a health questionnaire was administered by the project coordinator. The questionnaire was designed to obtain information on demographic, anthropometric and health related variables. After filling out of the questionnaire, participants were requested to donate at least 10 ml of blood. A computer generated, unique study code was assigned to every participant. Data collected from participants was saved in an excel sheet which was only accessible to the PI and project coordinator. The laptop computer was password protected and password was only provided to study coordinator and the Principal Investigator of the study. Confidentiality of all subjects was completely ensured.

### 2.3. Sample processing

A total of 10 ml of blood was drawn using 18-gauge needle and immediately transferred into a 15 ml conical tube containing 10 μls of heparin sulphate (heparin concentration is approximately 15 USP (US Pharmacopeia) units of heparin per milliliter of blood) to prevent coagulation. Blood was transported to the laboratory at the Department of Life Sciences, Lahore University of Management Sciences (LUMS), within 2 h of bleeding. Blood was processed as follows:

#### 2.3.1. Purification of peripheral blood mononuclear cells and granulocytes cells from whole blood

A total of 10 ml of whole blood collected from individuals with T2D or healthy volunteers, was layered on equal volume of a sucrose gradient, (Polymorphprep - Axis Shield UK Co) for separation of peripheral blood mononuclear cells (PBMC) and neutrophils (PMNs). The gradient in polymorphprep allows for the separation of PBMC and PMNs into two separate layers. PMNs obtained using this method are 99% pure. On average 4-6 × 10^6^ cells/ml RPMI were isolated from 10 ml of blood. Purified PMNs were re-suspended in 1 × PBS (pH 7.0) for the measurement of ROS and the remaining PMNs were re-suspended in assay medium (RPMI supplemented with autologous plasma) for use in NETs induction. PMNs were kept at room temperature and used the same day.

#### 2.3.2. Cells counting and cell number adjustment

Washed PMNs were re-suspended in RPMI (Sigma 1,640) and cell viability was determined using trypan blue solution (Gibco™, 0.4%), cells were suspended in RPMI+ 10 mM HEPES and 10% autologous human serum and were adjusted to the final concentration of 1×10^5^ PMNs/ml RPMI.

#### 2.3.3. Glucose treatment of neutrophils (PMNs)

For both ROS generation and induction of NETs, 1 × 10^5^ PMNs/ml RPMI were treated with 5 and 15 mM glucose for varying time intervals which are explained below alongside each experiment.

### 2.4. Advanced glycation end products preparation

Advanced glycation end products were generated by incubating bovine serum albumin (20 mg/ml) with 5 M D-Glucose and 0.2 M phosphate buffer (pH.7.4) for 60 days at 60°C under sterilized conditions. After incubation, amount of glycated BSA was 20 mg/ml. Sample was lyophilized, followed by dialysis against un-bound salt using Slide-A-Lyzer MINI dialysis device (Thermo-Fisher Scientific). The amount of glycated BSA was 18 mg/ml after removing the impurities. The glycated albumin was used in assays at a final concentration of 200 μg/ml.

### 2.5. Measurement of reactive oxygen species

A quantitative assay was performed to measure the generation of ROS in different conditions. ROS generated in response to glucose treatment at different time points was measured using Luminol/HRP chemiluminescence assay for ROS detection. Briefly, PMNs were seeded in 96-well black plate (Corning 3,991 polystyrene flat bottom) in triplicates and 50 μM Luminol (Sigma- Aldrich A4685) and 1.2 U/ml horseradish peroxidase (Sigma- Aldrich P8250) were added to each well. As a positive control, PMNs were stimulated with 600 nM PMA (Sigma P8139). Plates were gently tapped to ensure mixing of all reagents. Luminescence was immediately measured by taking multiple readouts for up to 5 min in a luminometer (Perkin Elmer).

### 2.6. Induction of NETosis in the presence and absence of glucose

To measure the effect of glucose on induction of NETs, purified PMNs were treated with different concentrations of glucose that mimics normoglycemic (5 mM = HbA1C 4.8%) and hyperglycemic conditions (15 mM = HBA1C 11%). Briefly, washed PMNs were re-suspended in buffer containing RPMI (Sigma 1640), 10 mM HEPES (H0887 Sigma-Aldrich) and 10% autologous heat inactivated serum and seeded into pre-made chambered slides coated with poly-l-lysine (0.1% (w/v) in H_2_O P8920 Sigma-Aldrich) at a concentration of 1 × 10^5^. PMNs were allowed to adhere to the wells for 30 min at 37°C in the presence of 5% CO_2_. Adherent PMNs were either incubated with (5 mM and 15 mM) glucose (158,968 Sigma-Aldrich) or with 600 nM PMA as a positive control for 4 h at 37°C in the presence of 5% CO_2_. To determine the impact of *in vivo* hyperglycemia on NETosis, PMNs isolated from those with T2D were incubated in the absence of glucose. Expression of NETs, was determined by staining extracellular DNA with propidium iodide (Invitrogen™ P3566). Cells were washed and slides were observed under confocal microscope (Nikon C1). Percentage of NETosis was determined by counting a total of sixteen fields per slide and averaging the counted fields.

### 2.7. Nets in hyperglycemia in the presence and absence of ROS inhibitor

The effect of high glucose mediated ROS on NETs generation was confirmed by using catalase (150 units) (Sigma-Aldrich), a specific inhibitor of hydrogen peroxide (H_2_0_2_). After isolation, 1 × 10^5^ cells/ml RPMI were seeded into 24 well plate on poly-l-lysine coated coverslips and stimulated with different glucose concentrations (5 mM, 15 mM) in the presence and absence of catalase (150 units). Stimulation of each concentration lasted for 4 h. Finally, the structure and location of NETs were observed under fluorescence microscopy. DNA quantitation was done using the standard protocol mentioned below.

### 2.8. Quantitation of NETs

Extracellular DNA was collected by scraping from the coverslip and solubilized using 20 units of DNAseI (Bio Basic DD0649), for 5 min at 37°C. Reaction was stopped by adding 0.05 M EDTA, extracellular DNA was quantitated using nano drop (Thermo scientific).

### 2.9. Measurement of RAGE, LCIII B, and neutrophil elastase using immunoblotting

Immunoblotting was used to measure expression of receptor for AGEs (RAGE), and intracellular markers of autophagy (LCIII B) and NETosis (neutrophil elastase). To determine the impact of hyperglycemia on NETosis and autophagy, PMNs isolated from those with poorly controlled diabetes were left stimulated. PMNs isolated from age and sex matched healthy volunteers were incubated in the presence or absence of 5 and 15 mM glucose for 30 and 120 min. On completion of incubation, samples were processed for western blotting according to the standard protocol. Briefly, glucose treated and untreated neutrophils were lysed in 2X laemmli sample buffer, sonicated (to shear DNA) at 50% amplitude for 15 s on ice, boiled at 95°C for 10 min, and loaded on to 12% (wt/vol) polyacrylamide gels for separation of proteins. Once the 6 kDa band of protein ladder (See Blue Plus-Invitrogen) reached the dye front at the end of the gel, the gels were removed from the cast and prepared for transfer to nitrocellulose membranes. Nitrocellulose membranes containing neutrophil proteins were blocked for 1 h in PBS containing 5% non-fat milk and 0.1% (wt/vol) Tween, to prevent non-specific binding of antibodies. After blocking, blots were washed and incubated with anti–neutrophil elastase (Santa Cruz) polyclonal antibody in 1:1000 dilution or anti–RAGE (Sigma-Aldrich) in 1:1000 or anti-LCIIIB (Abcam) or anti-GAPDH (santa-cruz) in 1:1000 dilution in PBST (Phosphate Buffered Saline pH7.4 + 0.1% Tween) containing 5% non-fat milk. Bound antibody was detected with enhanced chemiluminescence using horseradish peroxidase–conjugated anti–mouse-Ig secondary antibodies (southern biotech) in a dilution of 1:1000.

### 2.10. PMN activation and fractionation for measurement of activation of NADPH oxidase

PMNs isolated from patients with diabetes were not stimulated and processed directly. However, PMNs purified from non-diabetes individuals were stimulated with glucose (5 mM and 15 mM) with continuous shaking. After different time intervals, cells were pelleted and re-suspended in cell lysis buffer (10 mM PIPES [piperazine-N,N = -bis (2-ethanesulfonic acid)], pH 7.3, 100 mM KCl, 3 mM NaCl, 1.25 mM EGTA, 5 mM EDTA, 1 mM phenylmethylsulfonyl fluoride [PMSF], 20 g/ml leupeptin, 20 g/ml pepstatin). Samples were sonicated at 50% amplitude for 15 s on ice, and unbroken cells and nuclei were pelleted by centrifugation at 800 g for 10 min at 4°C. The supernatant was centrifuged at 50,000 ×*g* for 12 min at 4°C to pellet membranes. The pellet was re-suspended in solubilization buffer (20 mM Tris, pH 7.5, 1% SDS, 1 mM PMSF, 20 g mL leupeptin, and 20 g mL pepstatin) in a volume equal to the initial total volume. Cytosol and membranes (1 × 10^6^ cell equivalents) were incubated for 60 min on ice with solubilization buffer and then centrifuged at 15,000 *g* for 5 min to remove insoluble material. Equal volumes of each fraction, were mixed with 1X SDS sample buffer, and separated on 12% SDS-PAGE followed by transfer to polyvinylidene difluoride (PVDF) (Millipore) membrane. After transfer, membranes were blocked with 5% non-fat milk and 0.1% (wt/vol) tween, and probed with rabbit anti-p40phox (Santa Cruz), or mouse anti-glyceraldehyde-3-phosphate dehydrogenase (GAPDH) (Santa Cruz) at room temperature with continuous shaking. Blots were washed after 1 h with PBST and incubated with horseradish peroxidase labeled secondary goat anti- rabbit (Thermo Scientific) or anti-mouse IgG (southern biotech). Blots were developed with an ECL detection kit (GE Healthcare). Images were acquired on a Gel Doc System using Image Lab software (Bio-Rad).

### 2.11. NET detection by immunofluorescence

Poly-l-lysine coated coverslips were placed in a 24-well plate. PMNs were seeded at a concentration of 1 × 10^5^ cells/ml of RPMI on coverslips, stimulated, and exposed to NET-inducing stimuli. After formation, NETs were fixed with 4% PFA and incubated for 10 min at room temperature. Samples were washed twice with 300 μl sterile PBS for 10 min each time at room temperature. After washing, samples were permeabilized with 0.05% Triton X-100, and blocked using 300 μl blocking solution (2% BSA) for 30 min at room temperature. Primary antibody (anti-human neutrophil elastase) was added into samples and incubated overnight at 4°C in dark. Coverslips were washed thrice with sterile PBS for 5 min at room temperature. Fluorochrome-labelled secondary antibody diluted in blocking solution (2% BSA) was added to coverslips for 1 h at room temperature in dark. Coverslips were washed three times in PBS for 5 min at room temperature. Antibody solutions was aspirated and after washing, a 10,000-fold diluted DAPI was added to coverslips for 2 min at room temperature. Coverslips were washed twice in PBS for 5 min each time at room temperature to remove unbound secondary antibodies and DNA stain. Coverslips were dried on a paper towel, and carefully placed on clean, degreased microscope slide with 10 μl gold anti-fade mounting medium. The edges of the coverslip were sealed using sealing liquid and after drying observed under confocal microscope (Nikon C1).

### 2.12. LDH cytotoxicity assay

LDH Cytotoxicity assay was performed using Pierce LDH cytotoxicity assay kit (Thermo scientific 88,954) according to manufacturer’s instructions. Briefly, the optimal number of cells (1 × 10^5^ cells/ml) were seeded in 100 μl of RPMI medium in triplicate wells in a 96-well tissue culture plate. A complete medium control without cells was used to determine LDH background activity present in sera used for media supplementation. A serum-free media control was included to determine the amount of LDH activity in sera. Additional cells were plated in triplicate wells for spontaneous LDH activity controls (water) and maximum LDH activity controls (10X Lysis Buffer). The plate was incubated in an incubator at 37°C, 5% CO_2_ for 30 min. To the set of triplicate wells serving as the maximum LDH activity controls, 10 μl of lysis buffer (10X) was added, and mixed by gentle tapping. Different concentrations of glucose (5 mM and 15 mM), and inhibitors AZD6244 (10 μM), GDC0941 (2 μM), GDC0068 (2 μM), were added to cells. The plate was again incubated in an incubator at 37°C, 5% CO_2_ for 45 min. Each sample medium (50 μl) (e.g., complete medium, serum-free medium, Spontaneous LDH Activity Controls, glucose-treated, inhibitors-treated and Maximum LDH Activity Controls) was added to a 96-well flat-bottom plate in triplicate wells. Reaction mixture (50 μl) was transferred to each sample well and mixed using a multichannel pipette. The plate was incubated at room temperature for 30 min protected from light. Stop solution (50 μl) was added to each sample well and mixed by gentle tapping. The absorbance was measured at 490 nm and 680 nm. To determine LDH activity, the 680 nm absorbance value (background) was subtracted from the 490 nm absorbance before calculation of % cytotoxicity [(LDH at 490 nm) − (LDH at 680 nm)]. The % cytotoxicity was calculated as follows:

% Cytotoxicity = Glucose/inhibitors-treated LDH activity − Spontaneous LDH activity/Maximum LDH activity − Spontaneous LDH activity × 100.

### 2.13. Phagocytosis

In order to measure phagocytosis, PMNs were isolated using polymorphprep as described previously. Cell count was adjusted to 1 × 10^5^ cells/ml of RPMI. *Streptococcus pneumoniae* (D39 strain) were grown to an OD_600nm_ of 0.4. To measure phagocytosis in normoglycemic or hyperglycemic conditions, PMNs were incubated with *S. pneumonia* (multiplicity of infection 1:50) in the presence or absence of glucose (5 mM and 15 mM) for varying lengths of time (30 min, 60 min and 120 min). To allow for phagocytosis to occur, mixture containing pneumococci and PMNs were incubated at 37°C in a shaking incubator and samples were removed at pre-determined time points. After incubation, samples were centrifuged at 3000 rpm for 10 min at room temperature. Supernatant was discarded and pellets were washed twice with 1 X PBS. Ampicillin (10 μg/ml) was added to eliminate all the extracellular bacteria. PMNs were washed again to remove ampicillin with 1 X PBS, and lysed with 0.05% triton X-100 to release all the intracellular bacteria. The lysate was plated immediately on blood agar plates and left at 37°C incubator overnight in the candle jar. Next day the colonies were counted that gave an estimate of the phagocytosed bacteria.

### 2.14. Analysis of phagocytosis through FACS

The capsular type 2 strain of *S. pneumoniae*, D39, was grown to an OD_600nm_ of 0.4, centrifuged at 3,000 rpm for 10 min and labelled for 1 h at 4°C in PBS containing 250 μg/ml FITC. After labelling, bacteria were washed thoroughly to remove excessive dye and stored at −20°C. Isolated PMNs (2 × 10^6^ cells/ml) were incubated in different glucose conditions (5 mM and 15 mM) for 1 h at 37°C shaking incubator. Phagocytosis was performed in RPMI supplemented with 10% FBS, 10 mM HEPES and different concentrations of glucose. In a 24 well plate 100 μl of FITC labeled bacteria (4 × 10^8^ CFU/ml) were mixed with 1 ml of PMNs (2 × 10^6^ cells/mL) to reach a 20:1 MOI. The reaction was incubated for other 30 min at 37°C on a plate thermoshaker (170 rpm). Samples were then fixed for 1 h with 1 ml cold formaldehyde at the final concentration of 2%. Samples were acquired immediately after 1 h of fixation at 4°C and run on FACS Verse flow cytometer. PMNs without bacteria were gated to eliminate cell debris and measure their auto florescence on FITC-H to differentiate FITC+ PMNs in other samples. A total of 10,000 events were collected for each sample gated on neutrophils.

### 2.15. Statistical analysis

All experiments in this study were repeated in triplicates in at least three independent experiments. In case of patient samples, experiments were repeated on three different days with different diabetic samples. Statistical analysis was performed using GraphPad Prism 7 software and SPSS 22.0 (IBM, United States). Data are presented in the form of graphs and expressed as mean, ± standard error of the mean (SEM). Unpaired t-test, was performed to make comparison between two groups at one time point assuming unequal variances. One-way analysis of variance (ANOVA) was used to calculate differences between more than two groups. Two way ANOVA used to compare more than two groups with more than one time point. The criterion for statistical significance was taken as *p* < 0.05 (2 sided). Probability values of *p* < 0.05 and *p* < 0.001 were statistically significant.

## 3. Results

### 3.1. Demographic analysis of diabetes participants

The demographic and clinical characteristics of study participants are shown in Table 1. Table 2 categorizes those with diabetes on the basis of their most recent HbA1c values. A total of 60 cases were investigated during the study period, of which 30 individuals had diagnosed T2D whereas 30 were healthy controls. Most of the healthy controls were individuals visiting the outpatient clinic for complaints other than diabetes or were attendants of those with diabetes. The median age was 47 years for patients with T2D and 42.7 years for healthy controls. The male to female ratio was 43% males and 56% females among those with T2D, while in the healthy controls, 56% were males and 43% females. Among those with T2D, 23 out of 30 participants (76%) had a family history of diabetes, whereas nine participants (30%) in the non-diabetes group reported a family history of diabetes. Among those with T2D, 18 patients (60%) also had a history of hypertension; however, four (13%) non-diabetes participants reported a history of hypertension. A significant majority of those with diabetes were poorly controlled as suggested by their HbA1c values, where the lowest HbA1C value was 6.4 mM and the highest 14.1 mM with a mean HbA1C 10.15 ± 1.90. Similarly, individuals with T2D demonstrated high levels of random blood sugar (BSR), which ranged between 70 and 551 mg/dl, with a mean of 250 mg/dl. In comparison, BSR among the healthy controls was less than 110 mg/dl. None of these participants were diagnosed with tuberculosis or human immunodeficiency virus (HIV).

**Table 1:** Demographic and clinical variables of study participants.

Variables	Non-diabetes (*n* = 30) Mean ± SD	Diabetes (*n* = 30) Mean ± SD	*p* value
Age (years)	42.7 ± 9.37	47.0 ± 7.8	0.05
No (%male) No (% female)	17 (56%) 13 (43%)	13 (43%) 17 (56%)	0.30
Average neutrophil count	6 × 10^6^ cells/ml	4 × 10^6^ cells/ml	**<0.01**
BMI	29.0 ± 5.8	30.5 ± 6.6	0.47
HbA1c	5.36 ± 1.71	10.15 ± 1.90	**<0.001**
BSR	110	250.0 ± 115.8	-
HDL (mg/dl)	41.6 ± 12.2	72.5 ± 73.2	0.18
LDL (mg/dl)	103 ± 35.7	157.7 ± 64.0	**<0.01**
Cholesterol (mg/dl)	153.9 ± 28.5	195.3 ± 27.3	**<0.001**
Triglycerides	175.6 ± 138.6	280.4 ± 137.6	0.09
Hypertension	4 (13%)	18 (60%)	**<0.001**
Family history of DM	9 (30%)	23 (76%)	**<0.001**

Mean values with standard deviations presented in the table. Statistically significant *p* values are highlighted in bold.

**Table 2:** Patient distribution by HBA1c.

HBA1c groups	No. of subjects
6–8	08
9–11	17
12–14	05

### 3.2. PMNs isolated from individuals with T2D undergo spontaneous NETosis

**Figure 1:**
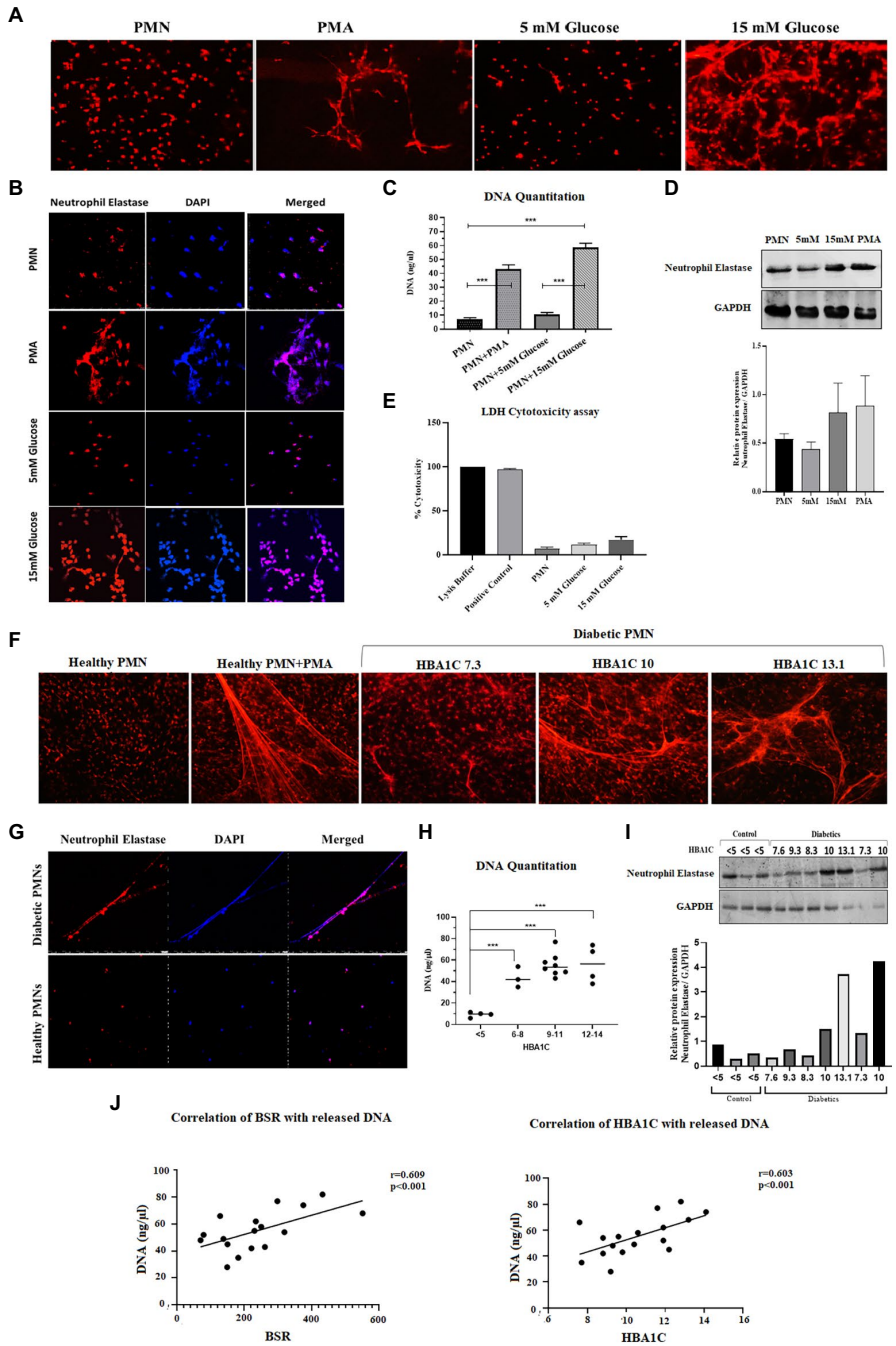
Spontaneous NETosis in PMNs isolated from individuals with T2D. **(A)** Fluorescent microscopy using propidium iodide DNA staining. The induction of NETosis was conducted on PMNs from healthy controls and treated with different concentrations of glucose (5 mM, 15 mM). PMA used as a positive control. The morphology of NETs observed after 4 h. Magnification: 20× **(B)** *in vitro* NETosis by immunofloresence for neutrophil elastase (red) and DAPI (Blue). **(C)** Quantification of DNA released during NETosis from neutrophils using nanodrop. Data were analyzed using one way ANOVA with multiple comparisons test for group wise comparison. *p <* 0.001 **(D)** Western blot showing levels of neutrophil elastase. PMNs isolated from healthy subjects were treated with different glucose concentration (5 mM and 15 mM) for 4 h and processed for western blotting. Lower panel showing GAPDH used as a loading control. Bar graph showing densitometry measurement of the ratio of neutrophil elastase/GAPDH (three representative blots from independent experiments). Columns are mean values; error bars are SEM **(E)** LDH cytotoxicity assay done to determine cellular cytotoxicity upon incubating healthy PMNs with 15 mM glucose. Graph shows hyperglycemic glucose concentration (15 mM glucose) does not cause cytotoxicity. **(F)** Diabetic PMNs exhibited increased spontaneous NETosis. Fluorescent microscopy using propidium iodide staining, indicative of increased spontaneous NETosis observed in PMNs isolated from representative patients with diabetes compared to healthy donors. Magnification: 20× **(G)** *in vitro* NETosis by immunofluorescence for neutrophil elastase (red) and DAPI (blue). Immunofluorescence Image showing the presence of neutrophil elastase on DNA strands. **(H)** Graph showing DNA Quantitation data using nanodrop. Data represented is indicative of three independent experiments. Data analyzed using one way ANOVA with multiple comparisons test. Data are represented as mean ± SE. *p* < 0.001. **(I)** Western blot showing basal level expression of elastase protein in PMNs isolated from diabetic subjects with different HbA1c values. PMNs from healthy subject with HbA1c less than 5 is taken as a control. Bar graph representing densitometry analysis of the ratio of neutrophil elastase/GAPDH. **(J)** Relationship of random blood glucose and percent glycated hemoglobin A1C (HbA1c) with DNA released by diabetic PMNs. Both random blood glucose (*r* = 0.609: *p* < 0.001) and HbA1c (*r* = 0.603: *p* = 0.01) showed a significant positive relationship with released DNA. Correlations were calculated using the Pearson’s correlation test.

Hyperglycemia has been shown to aggravate NETosis, indicating the significance of this event in the underlying aetiology of diabetes (40). We therefore wanted to measure NETosis in normoglycemic (5 mM = 4.8% HbA1c) and hyperglycemic (15 mM = 11% HbA1c) conditions. Incubation of neutrophils from healthy donors with 15 mM glucose for 4 h resulted in significant NETosis (Figure 1A). To confirm if extracellular structures represent NETs, immunofluorescence was done using antibodies to neutrophil elastase. Labeling for neutrophil elastase, when overlayed with DNA labeling (performed by DAPI), further confirmed that the extracellular matrix associated with neutrophils was indeed NETs (Figure 1B). Quantitation of DNA using nanodrop indicated that hyperglycemic conditions were associated with increased levels of extracellular DNA (58.64 ng/μl) as compared to normoglycemic conditions (10.52 ng/μl) *p* < 0.001 (Figure 1C). To further confirm if released DNA is the result of NETosis, we performed western blotting using antibodies to neutrophil elastase as a marker for NETs formation. An elevated level of protein was observed in neutrophils incubated with hyperglycemic conditions (15 mM glucose) as compared to normal glycemic conditions (5 mM glucose) or PMA. Densitometric analysis was performed to determine the ratio of neutrophil elastase/GAPDH from three independent experiments. Bar graph representing significantly elevated ratio of neutrophil elastase/GAPDH in the presence of 15 mM as compared to healthy PMNs (Figure 1D). To differentiate between cell lysis and NETosis, we performed lactate dehydrogenase assay. The presence of measurable quantities of extracellular lactate dehydrogenase (LDH), indicates the loss of viability of the cell and subsequent cell lysis. Low levels of LDH were identified associated with NETs suggesting the absence of cell lysis. LDH cytotoxicity assay has shown that neutrophils are not undergoing lysis upon incubation with 15 mM glucose which supports our hypothesis that the neutrophils are undergoing NETosis (Figure 1E).

In order to validate these findings *ex vivo*, PMNs were isolated from individuals with well-controlled (HbA1C 6–8%) or poorly controlled (HbA1C 9–14%) diabetes, where diabetes control was determined by HbA1c values. PMNs were incubated in the absence of a stimulus for 4 h, and NETosis was visualized as described in the material and methods section. PMNs isolated from individuals with poorly controlled T2D demonstrated NETosis in the absence of any stimuli. PMNs isolated from those with no diabetes showed very little NETosis. In order to obtain NETosis, comparable to those with poorly controlled diabetes, PMNs from those without diabetes had to be incubated with PMA (positive control; Figure 1F). Immunofluorescence showed neutrophil elastase associated with DNA strands indicating the extracellular DNA is the result of NETosis and not cell lysis in neutrophils from T2D subjects (Figure 1G). Quantitation of DNA using nanodrop indicated that hyperglycemic conditions were associated with increased levels of extracellular DNA as compared to normoglycemic conditions *p* < 0.001 (Figure 1H). NETosis was also confirmed by the presence of neutrophil elastase through western blotting Densitometry analysis showing significantly higher ratio of neutrophil elastase/GAPDH isolated from diabetic PMNs as compared to healthy PMNs (Figure 1I). Higher levels of neutrophil elastase were found associated with neutrophils from poorly controlled diabetes individuals, which correlated well with the DNA concentration and NETosis. The expression of GAPDH was used as a loading control. Next, we wanted to determine if there is a correlation between released DNA and blood glucose and HbA1c values. Results of Pearson Correlation demonstrated that there was a moderately positive (*r* = 0.6) association between blood glucose, HbA1c and release of DNA from diabetic neutrophils (Figure 1J).

### 3.3. Priming of neutrophils and subsequent NETosis in the presence of advanced glycation end products

**Figure 2:**
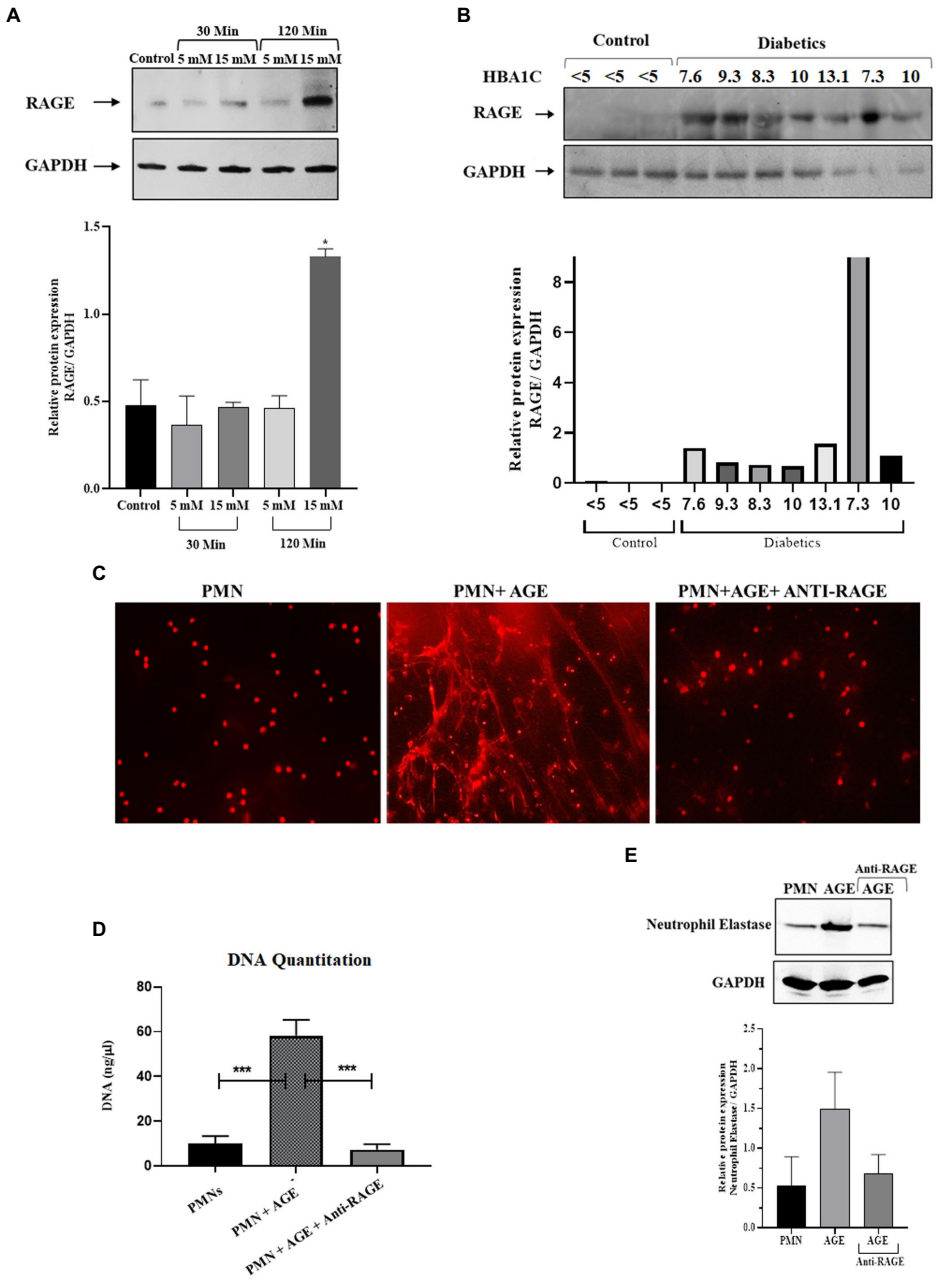
Priming of Neutrophils and subsequent NETosis in the presence of advance glycation end products. **(A)** Representative western blot analysis of RAGE expression in PMNs. Cells were treated with various concentrations of glucose (5 mM and 15 mM) for varying time points (30 min and 120 min). After given time points samples were prepared according to the standard protocol. A total of 10 μg protein loaded onto each well. Hyperglycemic glucose concentration (15 mM) leads to elevated expression of RAGE after 120 min of incubation. GAPDH used as a loading control. Bar graphs showing densitometric analysis of ratio of RAGE/GAPDH using two independent blots from two different experiments. Columns are mean values; error bars are SEM. Asterisks show level of significant difference from basal *p < 0.01 **(B)** Western blot showing RAGE protein expression in PMNs isolated from diabetic subjects with different HbA1C values. Healthy subjects with HbA1c value <5 are used as control. RAGE; receptor for advanced glycation end products. Bar graph representing densitometric measurements of RAGE/GAPDH in diabetic PMNs. Experiments were performed thrice, with data from a representative experiment shown **(C)** Representation of *in-vitro* NET release. Indicative of increased NETosis observed in PMNs isolated from representative healthy donors and incubated with AGE for 4 h. NETosis was decreased in the presence of Anti-RAGE antibody **(D)** Graph showing DNA Quantitation data using nanodrop. Data are represented as mean ± SE. ***p < 0.001. **(E)** Western blot showing levels of neutrophil elastase protein expression after incubating cells with AGE. Lower panel showing GAPDH as a loading control. Bar graph showing densitometry analysis of neutrophil elastase/GAPDH (two independent experiments). Columns are mean values; error bars are SEM.

Hyperglycemia has been shown to impact cellular functions in multiple ways (41). One of the most common mechanisms is *via* glycation of various structural and functional proteins, thereby resulting in the generation of advanced glycation endproducts or AGE (42). These glycated proteins bind to their cognate receptors called Receptors for Advance Glycation End Products or RAGE. The RAGE receptor is a scavenger receptor and is ubiquitously expressed on endothelial cells, including immune cells, such as macrophages and neutrophils (43). Therefore, in addition to hyperglycemia, we wanted to determine the impact of downstream products of hyperglycemia, such as AGE, on the formation of NETs. As demonstrated previously, the AGE binds to RAGE and that presence of AGE increases the expression of RAGE on the surface of PMNs. We first wanted to determine if there is an increase in the expression of RAGE on the surface of PMNs isolated from those with diabetes. Additionally, we also wanted to determine if transient acute glycemia, in response to incubation of healthy PMNs in hyperglycemic conditions, would lead to the expression of RAGE. Our results indicated that incubation of PMNs with 15 mM glucose for 120 min led to increased surface expression of RAGE, whereas low levels of hyperglycemia or incubation of PMNs with high levels of glucose for a short period of time did not impact RAGE levels on the surface of PMNs. Densitometry analysis showing relative protein expression of RAGE/GAPDH (Figure 2A). Next, to determine if PMNs isolated from those with different levels of glucose control, would also show differences in RAGE levels, we performed similar measurements on PMNs isolated from those with diabetes showing varying levels of HbA1c. Interestingly we observed differences in levels of RAGE associated with PMNs, where highest levels of RAGE expression was observed in individuals with HbA1c ranging from 7 to 10. Control non-diabetes individuals had very little or non-detectable levels of surface RAGE confirmed through densitometry analysis of RAGE/GAPDH (Figure 2B).

In order to determine if binding of AGE to RAGE leads to NETosis, we performed assays to detect NETS generation. We observed extensive NETosis of neutrophils in the presence of AGE as compared to absence of AGE (Figure 2C) which was further confirmed by DNA quantitation using nanodrop (Figure 2D) and release of neutrophil elastase in the presence of AGE (Figure 2E). Western blot showing that the release of neutrophil elastase by AGE was blocked by incubation with the anti-RAGE antibody confirming the specificity of AGE action. Densitometry analysis demonstrating the relative protein expression of RAGE/GAPDH in response to AGE in the presence and absence of anti-RAGE antibody.

### 3.4. Increased levels of ROS are associated with T2D

**Figure 3:**
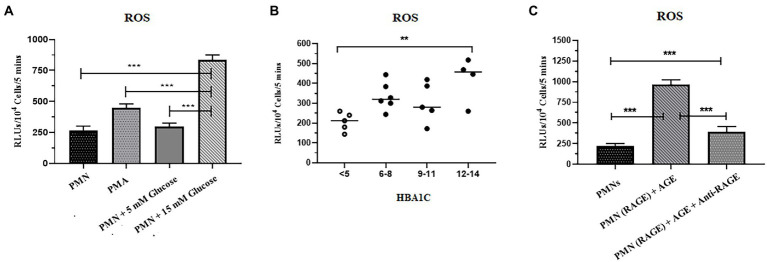
Increased levels of ROS were associated with T2D. **(A)** ROS generation was quantified in response to glucose in healthy peripheral blood neutrophils (PMNs) using luminol HRP assay. PMNs were incubated in 5 mM glucose (physiological concentration) and 15 mM glucose (high glucose concentration) and RLUs measured for upto 5 min in luminometer. Bar graph showing data from three independent experiments. Data analyzed using one way ANOVA with multiple comparisons. *p* < 0.001 **(B)** Total reactive oxygen species produced by PMNs of non-diabetic and diabetic subjects. PMNs isolated from non-diabetic individuals and diabetic patients subjected for ROS secretion at resting condition. The maximal production of ROS at any time point over a period of 5 min was recorded and compared using one way ANOVA. PMNs isolated from diabetic patients showed a higher and consistent secretion of ROS in resting condition. Data expressed as mean ± SEM and the statistical significance was determined at *p* ≤ 0.01. ROS: Reactive oxygen species; RLUs: Relative luminisence units. **(C)** Levels of ROS generated after incubating PMNs with AGE in the presence and absence of anti-RAGE antibody. Significantly elevated levels of ROS were recorded in the presence of ROS as compared to healthy control and AGE in the presence of anti-RAGE antibody. Data analyzed using one way ANOVA with multiple comparison test for comparing different groups. Statistical significance was determined at *p* < 0.001.

Reactive oxygen species serves as a second messenger for the generation of NETs (44). We, therefore, tested the hypothesis that hyperglycemia might prime PMNs to produce elevated levels of ROS, which leads to spontaneous NETosis in the absence of second stimuli. The levels of ROS in PMNs isolated from healthy volunteers in normoglycemia and hyperglycemic conditions were measured. For that, PMNs isolated from the healthy subject were incubated with 5 mM (physiological concentration) and 15 mM (hyperglycemic concentration) glucose for 30 min. Phorbol myristate acetate (PMA), known to induce ROS *via* activation of NADPH, was used as a positive control. Levels of ROS were measured using Luminol/HRP assay according to the standard protocol. Resting PMNs isolated from healthy donors demonstrated low levels of ROS, which did not change significantly in the presence of normoglycemic conditions. However, up to 3 fold increase in ROS was observed in PMNs incubated with 15 mM of glucose, indicating that hyperglycemia increases levels of ROS, which was significantly higher compared to normoglycemic condition (*p* < 0.001) (Figure 3A).

To validate these findings further, we isolated PMNs from those with T2D with HbA1c values, ranging from 6 to 14%, and measured levels of ROS generated intrinsically. Samples collected from those with poorly controlled diabetes demonstrated significantly elevated levels of ROS as compared to healthy PMNs (*p* = 0.01; Figure 3B).

While hyperglycemia results in AGing of proteins and lipids, binding of AGE to RAGE than continues the cycle of ROS generation and subsequent oxidative stress (42). We thus determined if the binding of AGE to RAGE is also impacting the levels of ROS in healthy PMNs. Incubation of healthy PMNs with AGE for 30 min demonstrated a significant increase in levels of ROS (*p* < 0.001; Figure 3C). To demonstrate that the observed increase in the levels of ROS were resulting from the interaction between AGE and RAGE, samples were incubated for another 30 min before measuring levels of ROS. A significant decrease (60%) in the levels of ROS was observed in the presence of antibodies to RAGE, indicating that binding of AGE to RAGE is associated with an increase in levels of ROS (*p* < 0.001) (Figure 3C).

### 3.5. Elevated levels of ROS are associated with the activation of NADPH oxidase in hyperglycemic conditions

**Figure 4:**
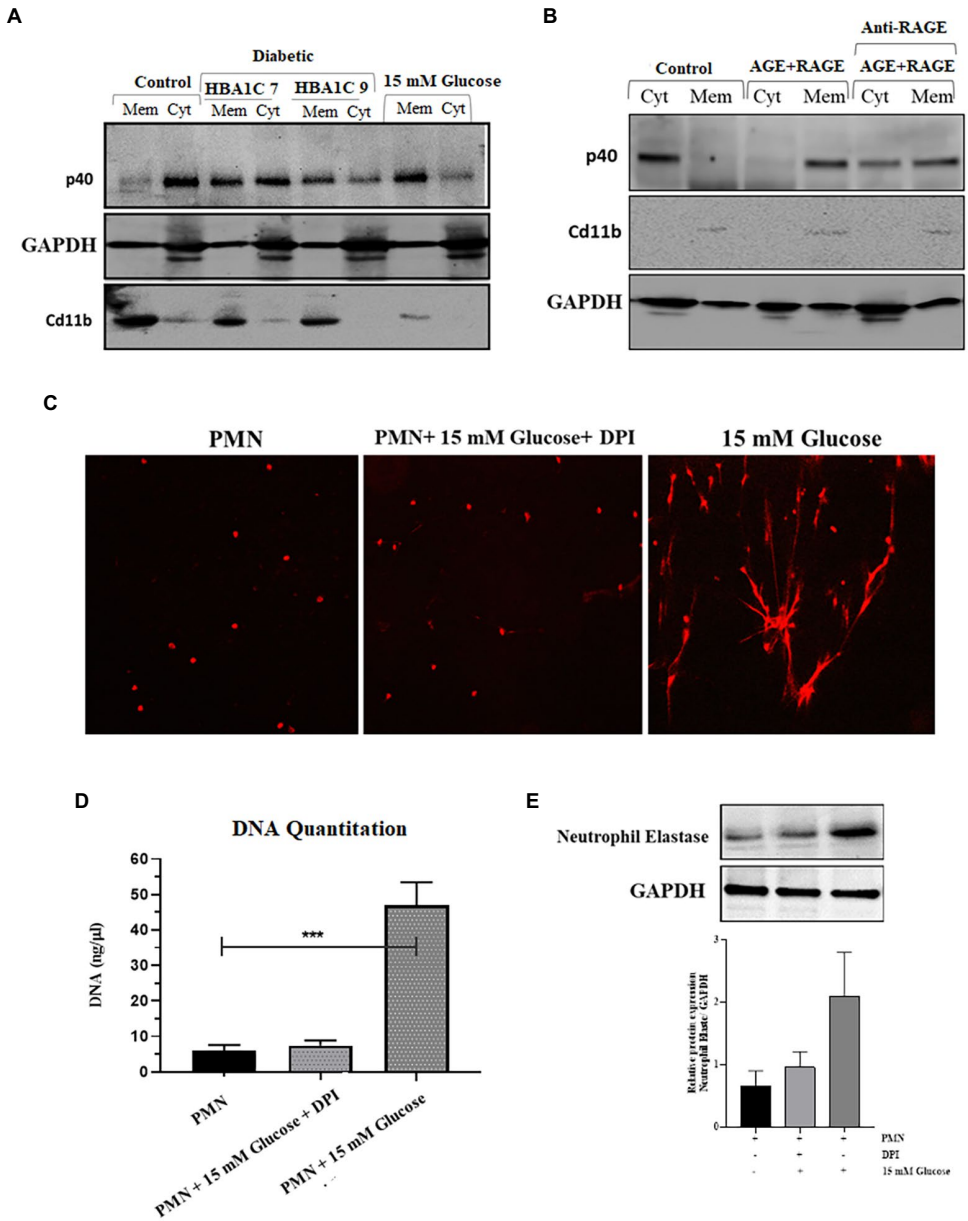
Elevated levels of ROS are associated with activation of NADPH oxidase in hyperglycemic conditions. **(A)** Western blot showing translocation of NADPH oxidase p40phox cytoplasmic subunit to neutrophil membranes. Primary human neutrophils (PMNs) isolated from healthy subjects were incubated with 15 mM glucose for 120 min. PMNs isolated from diabetic individuals with different HbA1C (7 and 9) were not stimulated with glucose. PMNs were separated into membrane and cytosol fractions, and the amounts of p40phox in neutrophil cytosol and membrane fractions were analyzed by western blotting. The membrane-associated Cd11b protein served as a loading control for membrane fractions and GAPDH as a control for cytosolic fraction. The results presented are representative of three independent experiments. **(B)** Western blot showing activation of NADPH oxidase complex as indicated by translocation of p40phox subunit from cytosol to membrane fraction after incubating healthy PMNs with AGE (200 μg/ml) in the presence and absence of anti-RAGE antibody. Membrane associated Cd11b protein is used as a control for membrane fraction. GAPDH used as a loading control for cytosolic fraction **(C)** Florescence microscope images showing NETS formation in the presence and absence of NADPH oxidase inhibitor, Diphenylene ionodonium (DPI) (10 μM) confirming the role of NADPH oxidase generated ROS in the process of NETs generation. **(D)** DNA quantitation showing significantly decreased levels of ROS in the presence of DPI. One way ANOVA used to compare groups. ***p < 0.001. **(E)** Western blot showing the decreased expression of neutrophil elastase protein in the presence of DPI as compared to 15 mM glucose. Lower panel showing GAPDH used as a loading control. Bar graph representing densitometric measurements of ratio of neutrophil elastase/GAPDH using two independent blots. Columns are mean values; error bars are SEM.

NADPH oxidase is a multicomponent enzyme system (45). In resting cells, this enzyme system is not activated (not assembled), and the components are divided between the membrane and the cytosol. The neutrophil NADPH oxidase is comprised of plasma membrane-bound subunits (gp91phox and p22phox) comprising flavoCytochrome b 558 and cytosolic subunits (p47phox, p67phox, p40phox, and Rac2). The activation of PMNs by stimuli, such as fMLP or PMA, causes the phosphorylation and translocation of the cytosolic components to the plasma membrane, where they interact with flavoCytochrome b 558. NADPH oxidase activation can result from either an increase in the expression of one or several subunits or by translocation of cytosolic subunits to the plasma membrane (46). We, therefore, wanted to test the hypothesis that ROS production as observed in the previous section (Figure 3A), is the result of the priming or partial activation of NADPH oxidase in response to hyperglycemic conditions. To confirm the activation of NADPH oxidase complex, we measured the expression and translocation of p40 (cytosolic subunit), which when active, translocates from the cytosol to the membrane. Our results indicated that in resting PMNs, obtained from healthy individuals, p40 was predominantly cytoplasmically located; however, in hyperglycemic conditions (15 mM), partial translocation of p40 subunit from cytosolic to membrane fraction was observed in 120 min post glucose exposure. CD11b, a membrane receptor, was used as a control for membrane fraction, and GAPDH was the control for the cytosolic fraction. To determine if hyperglycemic conditions in those with poorly controlled diabetes are also associated with translocation of p40 subunit and subsequent activation of NADPH in PMNs, we measured translocation of p40 subunit in PMNs isolated from individuals with T2D. Fractionation of PMNs isolated from those with T2D, demonstrated an increased membrane localization of p40 subunit compared to the PMNs from healthy individuals, indicating that the NADPH oxidase complex was primed in those with T2D (Figure 4A).

We next determined if the binding of AGE-RAGE would activate NADPH-oxidase, a multiprotein enzyme complex responsible for the activation and release of ROS from PMNs. PMNs isolated from healthy donors were incubated with 200 μg/ml AGE for 120 min, and membrane localization of p40 subunit was measured. Our results demonstrated that in PMNs incubated with glycated BSA, p40 subunit translocated to the cytoplasm after 120 min incubation (Figure 4B), whereas in the presence of antibody to RAGE, we found p40 to be both membrane and cytoplasmically located.

Next, we wanted to determine if ROS generated in response to priming of PMNs by activation of NADPH results in induction of NETs generation. For that, we blocked the activation of the NADPH oxidase complex by using Diphenyleneiodonium (DPI), a potent inhibitor of flavoenzymes. Our results indicated that pretreatment of PMNs with DPI (10 μM) for 1 h blocked NETs formation, despite activation with 15 mM glucose (Figure 4C); this was further confirmed by DNA quantitation (*p* < 0.001; Figure 4D). Western blot also confirmed that after blocking NADPH oxidase complex with DPI, the level of neutrophil elastase protein complex is decreased as compared to 15 mM glucose. Densitometry measurements showing relative protein expression of neutrophil elastase/GAPDH in the presence and absence of DPI (Figure 4E).

### 3.6. Elevated levels of ROS and subsequent NETosis are associated with poorly controlled T2D

**Figure 5:**
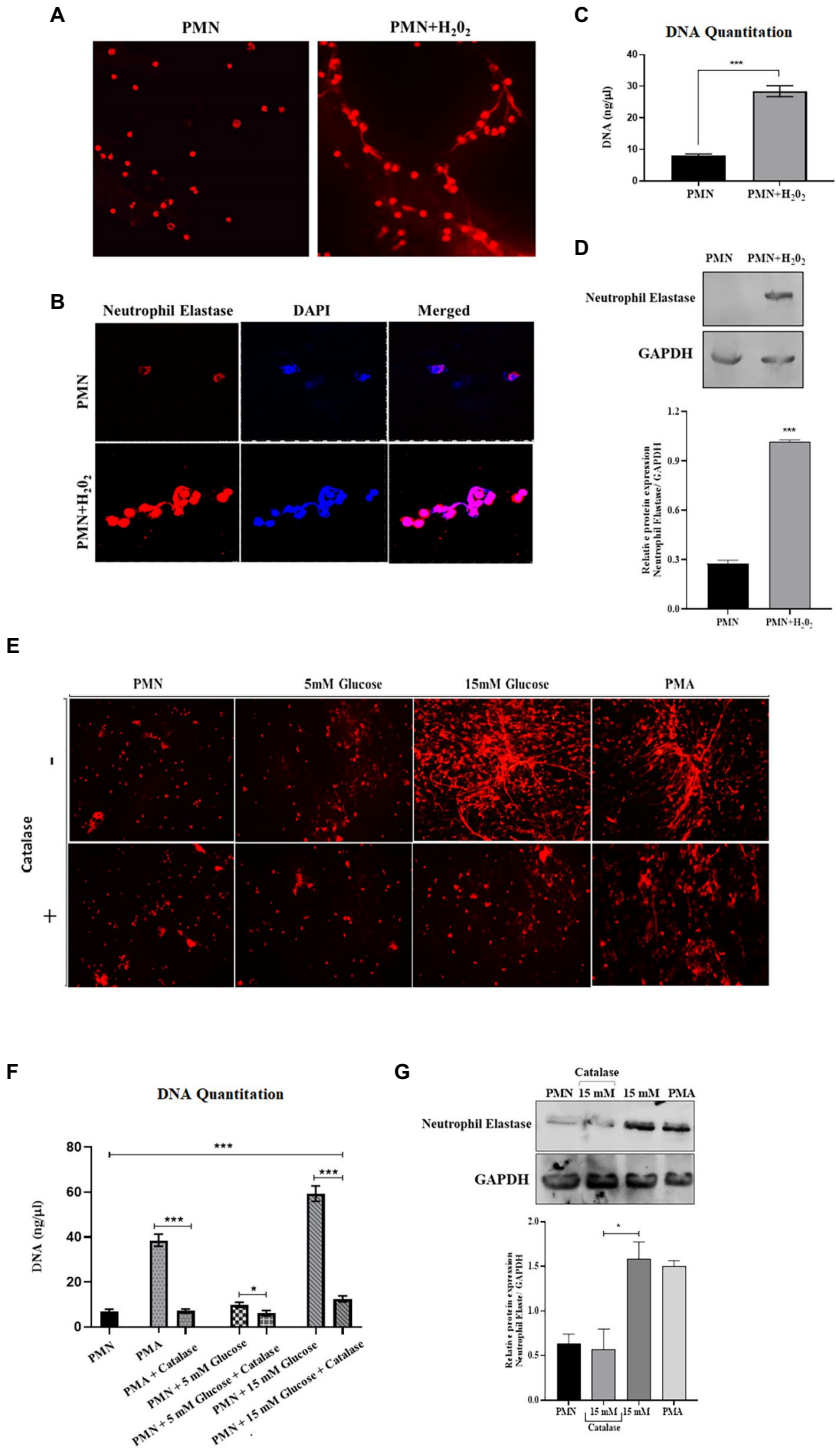
An association was observed between elevated levels of ROS and NETosis in poorly controlled T2D. **(A)** Representative images of NETs released from PMNs stimulated with H_2_0_2_ (100 μM), and unstimulated control. Results are representative of three independent experiments. Magnification 20×. **(B)** Immunofluorescence image showing association of neutrophil elastase (red) with DNA stained with DAPI (blue). **(C)** DNA quantitation values using nanodrop. Bar graph representing data from three independent experiments. Data analyzed using unpaired T-test. Data expressed as mean ± SEM and the statistical significance was determined at *p* ≤ 0.001. **(D)** Western blot showing the levels of neutrophil elastase upon stimulating healthy PMNs with hydrogen peroxide (100 uM) for 4 h. Bar graph showing densitometric analysis of neutrophil elastase/GAPDH from two independent experiments. Column represents mean values while error bar represents SEM **(E)** Antoixidant block NETs production *in vitro*. The induction of NETosis was conducted on PMNs from healthy controls and treated with PMA, 5 mM and hyperglycemic concentrations of glucose (15 mM) in the presence and absence of antioxidant (catalase, 150 units) for 4 h. **(F)** DNA quantitation using nanodrop. Data analyzed using one way ANOVA with multiple comparisons. Data expressed as mean ± SEM and the statistical significance was determined at p value ≤0.001. **(G)** Western blot showing the levels of neutrophil elastase protein expression after incubating neutrophils with 15 mM glucose in the presence and absence of catalase. PMA used as a positive control. Lower panel showing GAPDH as a loading control. Bar graph showing densitometric analysis of ratio of neutrophil elastase/GAPDH from three independent experiments. Column represents mean: error bar represents SEM. *p < 0.01.

To explore the relationship between ROS production and NETs extrusion, PMNs were isolated from healthy subjects and NETs were generated using hydrogen peroxide (H_2_0_2_) (100 μM), the well-established ROS. PMNs were seeded into wells in the presence or absence of hydrogen peroxide. When DNA is extruded extracellularly, it is bound by propidium iodide and fluoresces when excited. After 4 h, the samples were fixed and mounted and observed in a fluorescence microscope to morphologically confirm the presence of NETs. As little as 100 μM H_2_0_2_ was sufficient to induce PMNs to release extracellular structures that fluoresced in the presence of propidium iodide (Figure 5A). When observed through immunofloresence, neutrophil elastase appeared to be associated with DNA strands, confirming the release of NETs (Figure 5B) and quantitated using nanodrop (Figure 5C). Neutrophil extracellular traps (NETs), extruded from neutrophils upon activation, has been shown to be dependent on reactive oxygen species (ROS) generation. However, NET release can also occurs through a rapid ROS-independent mechanism (47). Thus, to examine the requirement of ROS in the generation of NETs and to confirm that these assays were compatible with detecting ROS-dependent release of NETs, western blotting was performed to check the levels of neutrophil elastase protein expression in the presence and absence of hydrogen peroxide (H_2_0_2_). An elevated level of neutrophil elastase expression was observed in the presence of ROS as depicted by densitometry analysis (Figure 5D). We further aimed to block ROS, particularly hydrogen peroxide using catalase, to confirm if the inhibition of pathways involved in ROS generation leads to reduction of NETs formation. For that, we treated PMNs isolated from a healthy volunteer with different concentrations of glucose (5 mM and 15 mM) in the presence and absence of catalase (150 units), a potent inhibitor of hydrogen peroxide (H_2_0_2_). NETs generation assay was performed to qualitatively visualize NETs using a florescence microscope. Our results demonstrated that the addition of antioxidant catalase significantly reduced the release of NETs even in the presence of hyperglycemic glucose concentration (Figure 5E). We further quantified DNA released during NETs generation using nanodrop. We observed a significant decrease (*p* < 0.001) in the levels of released DNA after the addition of antioxidant (12.75 ng/μl) as compared to 15 mM glucose (61.5 ng/μl; Figure 5F), suggesting a role of ROS in the induction of spontaneous NETosis in those with T2D. Western blot analysis demonstrated lower expression of neutrophil elastase in the presence of an antioxidant, further demonstrated by densitometry analysis showing relative protein expression of neutrophil elastase/GAPDH (Figure 5G).

### 3.7. Elevated levels of ROS impair the phagocytic ability of PMNs in T2D

**Figure 6:**
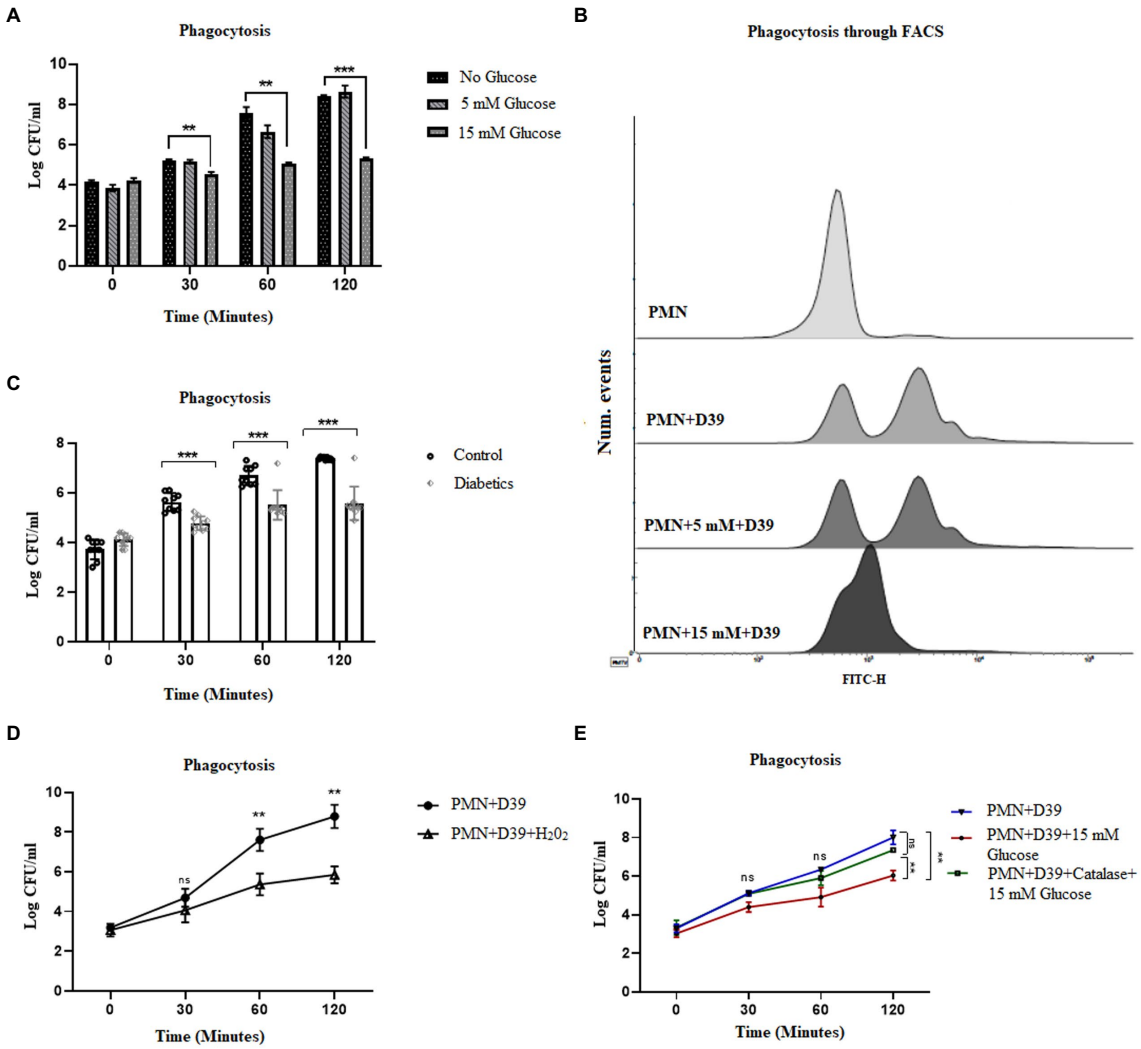
Hyperglycemia mediated ROS impairs phagocytic ability of PMNs in T2D. **(A)** Analysis of neutrophil phagocytosis in the presence of different glucose concentrations. Percentage of PMNs phagocytically active as determined by the percentage of PMNs which have phagocytosed *S. pneumonia* (D39 strain). Log CFUs of ingested *S. pneumoniae* as an indication of neutrophil phagocytic activity at different time points and at different glucose concentration. Bar graphs representing data from three independent experiments. Data analyzed using two way ANOVA with multiple comparison test. Data expressed as mean ± SEM and the statistical significance was determined at p value ≤0.001 **(B)** Analysis of phagocytosis through FACS. Uptake of FITC-labelled D39 strain of *S. pneumonaie* by healthy PMNs. PMNs isolated from healthy controls were incubated with 5 mM and 15 mM glucose for 1 h and incubated with FITC-labelled D39 for 30 min at 37°C. Phagocytic activities were analyzed using flow cytometry. Data are presented as histograms. **(C)** Phagocytosis of *S. pneumonia* (D39 strain) by human PMNs (diabetics vs. healthy controls) for 2 h. PMNs were isolated from diabetics and healthy individuals and incubated with *S. pneumoniae* for 2 h. At each time point diabetic PMNs showed significantly reduced phagocytosis activity as compared to healthy controls. Statistical significance was determined by comparing samples between healthy and diabetics at different time points (two-way ANOVA, Bonferroni) with significance *p* < 0.001. **(D)** Phagocytosis of *S. pneumoniae* by human PMNs in the presence and absence of hydrogen peroxide (H_2_0_2_) (100 μM) for 2 h. In the presence of hydrogen peroxide (H_2_0_2_), phagocytosis was decreased significantly. Data expressed as mean ± SEM and the statistical significance was determined at p value ≤0.01. **(E)** Phagocytosis of *S. pneumoniae* by human PMNs in the presence and absence of antioxidant catalase (150 units) and 15 mM glucose for 2 h. Graph represent results of three independent experiments. Data analyzed using two way ANOVA with multiple comparison test.

While NETosis, helps to contain the infection, phagocytosis remains an important killing mechanism employed by PMNs (48). Reactive oxygen species have been shown to be important for both NETosis and phagocytic killing (49). However, it remains to be determined if elevated levels of ROS oscillate the bactericidal activity towards NETosis or phagocytosis. To determine how hyperglycemia impacts the phagocytic ability of PMNs to engulf and kill *S. pneumoniae*, healthy PMNs were incubated with hyperglycemic conditions followed by incubation with *S. pneumoniae*. Aliquots were removed at varying time intervals to measure phagocytosis, as explained in the material and methods section. Our results (Figure 6A) showed that compared to hyperglycemic conditions where the phagocytic ability of PMNs decreased overtime, optimum phagocytic activity of peripheral PMNs was observed in the absence of glucose and in the presence of normoglycemic conditions. To determine whether the phagocytosed bacteria were viable, the number of viable bacteria were determined by colony forming unit (CFU) analysis. Compared to the no glucose controls, the relative CFU measured at 15 mM glucose were significantly lower when incubated for 2 h (Figure 6A) as compared to no glucose or normoglycemic conditions (5 mM). In order to validate these findings, we further quantified levels of phagocytosis through FACS. Our results demonstrated that the ability of healthy PMNs to phagocytose fluorescently labeled D39 strain of *S.pneumoniae* after stimulating with high glucose (15 mM) was markedly decreased as compared to no glucose and 5 mM glucose controls. Thus, confirming that the observed decrease in the number of colonies in Figure 6A represents impaired phagocytosis of bacteria in the presence of high glucose (Figure 6B). Diabetic PMNs were also assessed for their ability to phagocytose *S. pneumoniae ex vivo*. PMNs isolated from those with diabetes showed decreased phagocytosis as suggested by less intracellular bacteria compared to healthy PMNs (Figure 6C). At each time point, the ratio of extracellular to intracellular *S. pneumoniae* was very high in PMNs isolated from those with diabetes as compared to age and sex matched controls (*p* < 0.001), suggesting a markedly reduced phagocytic capacity of PMNs isolated from those with diabetes.

While ROS is responsible for the killing of bacteria during the process of phagocytosis, excessive production of ROS, as observed in diabetes, has been shown to impair bacterial phagocytosis and intracellular killing (50). To determine if ROS generated in hyperglycemic conditions is associated with impairment in phagocytosis, we used hydrogen peroxide H_2_0_2_ (100 μM). Incubation of PMNs with capsule type 2 strain of *Streptococcus pneumoniae* D39_,_ in the presence of H_2_0_2_, showed a decrease in the phagocytosis of *S. pneumoniae*. However, in the absence of H_2_0_2,_ the phagocytosis increased and peaked in 2 h, further confirming our hypothesis that hyperglycemia-mediated ROS impairs phagocytosis and intracellular killing of bacteria (Figure 6D). Furthermore, we measured the phagocytosis *of S. pneumoniae* in PMNs treated with catalase (150 units). Our results indicated that contrary to what we observed in NETosis, phagocytosis of *S. pneumoniae*, was restored in the presence of catalase. We started to observe an increase in the number of intracellular colonies at all-time points in catalase treated PMNs, suggesting an increase in the number of phagocytosed bacteria (Figure 6E).

### 3.8. ROS generated through hyperglycemia and AGE/RAGE signaling induces autophagy that leads to NETosis

**Figure 7:**
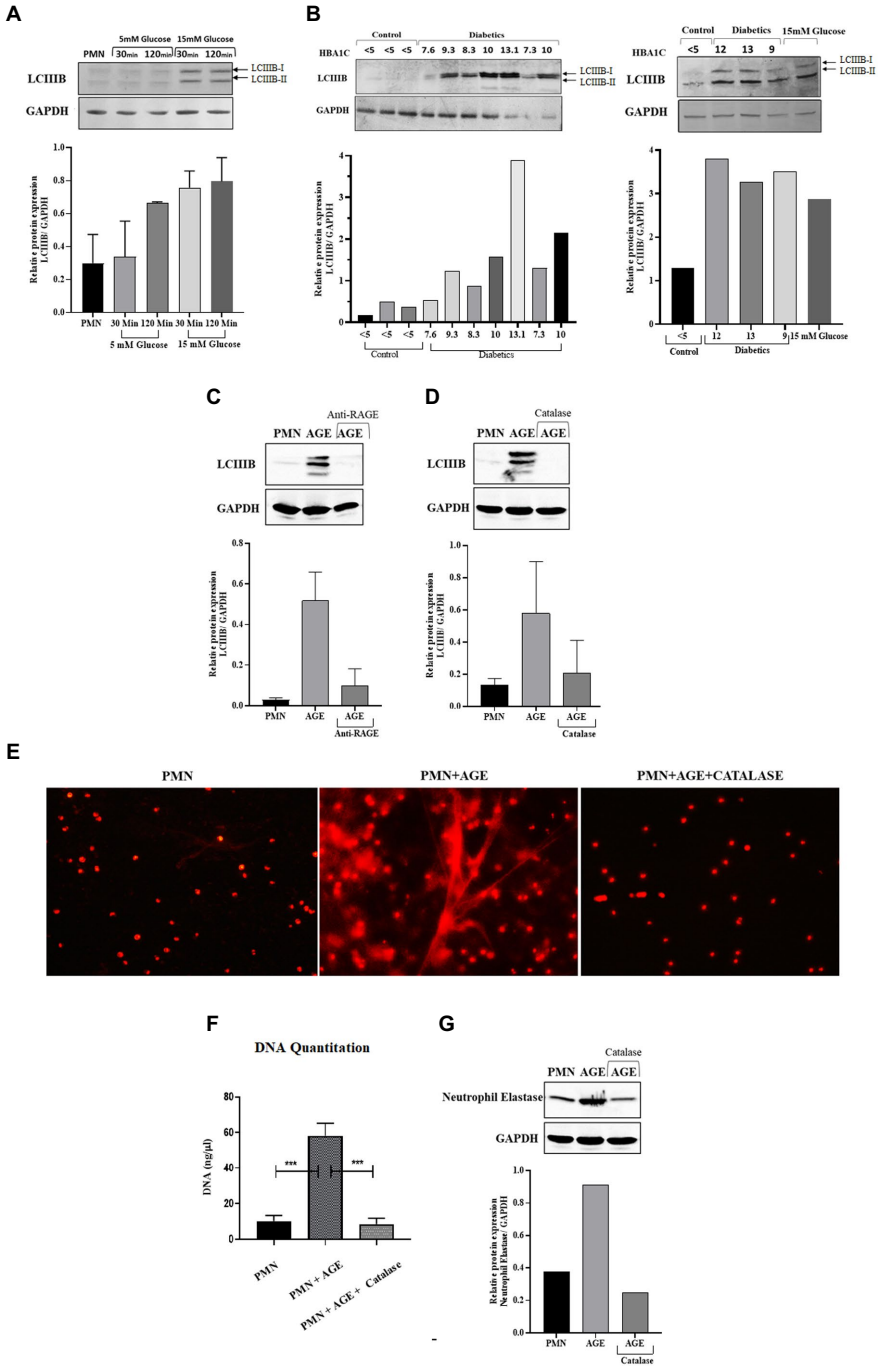
ROS generated through hyperglycemia and AGE/RAGE signaling induces Autophagy that leads to NETosis: **(A)** Western blot analysis of LCIIIB I and LCIIIB II proteins levels in human PMNs isolated from healthy individuals. Cells were incubated with different glucose concentrations (5 mM and 15 mM) for varying time points (30 min and 120 min). Where 15 mM glucose resulted in enhanced expression of LCIIIB protein after the given time points. Bar graphs representing densitometry measurements of ratio of LCIIIB/GAPDH (two independent experiments). Column represents mean, error bar represents SEM **(B)** Western blot showing basal level protein expression of LCIIIB I and LCIIIB II proteins in PMNs isolated from diabetic individuals with different HbA1C values mentioned. PMNs from healthy individual with HBA1c values <5 were used as healthy control. Lower panel showing GAPDH as a loading control. Western blots are representative of three independent experiments (n = 3). Densitometry analysis represented using bar graph. **(C)** Representative western blot analysis of LCIIIB I and LCIIIB II levels in human PMNs pre-treated as indicated with AGE (200 μg/ ml) for 120 min and anti-RAGE antibody as compared to untreated cells. Incubation with AGE lead to enhanced expression of LCIIIB protein. GAPDH used as a loading control. Densitometry analysis of the autophagy signal (LCIIIB) in healthy PMNs treated with AGE in the presence and absence of anti-RAGE antibody. Columns are mean values of LCIIIB/GAPDH; error bars are SEM. Asterisks indicates significant reduction in autophagy in the presence of anti-RAGE antibody. **(D)** Representative western blot image of LCIIIB I and LCIIIB II protein using human PMNs treated with AGE in the presence and absence of antioxidant catalase (150 units). Cells left untreated were used as a control. Lower panel showing GAPDH used as a loading control. Bar graph showing densitometry analysis of ratio of LCIIIB/GAPDH from two independent experiments **(E)** Florescence microscopic images representing *in-vitro* NET release. Increased NETosis observed in PMNs isolated from healthy subjects incubated with AGE for 4 h. NETosis was decreased in the presence of antioxidant catalase (150 units). **(F)** DNA Quantitation data using nanodrop. Bar graphs represent mean DNA release from three independent experiments. Error bars represent SEM. Data analyzed using *t*-test. Asterisks represent significant difference of DNA release. ***p < 0.001. **(G)** Western blot showing levels of neutrophil elastase protein expression after incubating cells with AGE in the presence and absence of catalase. Lower panel showing GAPDH as a loading control. Bar graph representing ratio of neutrophil elastase/GAPDH from representative experiment.

Reactive oxygen species have been shown to modulate several cellular processes. One such process is autophagy, which is a mechanism of self-sustenance (24, 25). In the previous section, we observed inhibition of phagocytosis by blocking ROS. Since ROS plays an important role in NETosis, phagocytosis and autophagy, we next wanted to determine how blocking ROS would impact autophagy. As autophagy is an important cellular pathway that facilitates both NETosis and phagocytosis, it was therefore imperative to address the following scenarios (i) how hyperglycemia modulates autophagy (ii) does modulation of autophagy impact the bactericidal activity of neutrophils (iii) which bactericidal process (phagocytosis and NETosis) is most impacted. To investigate the effects of ROS on autophagy, and its downstream impact on phagocytosis and NETosis, PMNs from healthy individuals were subjected to glucose induction using 5 mM and 15 mM of glucose. PMNs were exposed to each concentration of glucose for 30 min and 120 min. At the end of each incubation, we measured LCIIIB using western blotting. An increase in levels of LCIIIB was observed, indicating induction of autophagy at 15 mM at both 30 min and 120 min time points as shown by densitometry measurements (Figure 7A). In comparison to induced PMNs, we observed basal levels of LCIIIB in PMNs incubated in the absence of glucose or in the presence of 5 mM (normoglycemic conditions) of glucose. Next, we compared levels of LCIIIB in individuals with diabetes and age and sex matched controls. As shown in Figure 7B, we observed significantly elevated levels of LCIIIB in individuals with diabetes which correlated with their blood glucose control as indicated by HbA1c values. Basal levels of LCIIIB were observed in age and sex matched controls and in individuals with well controlled diabetes. Our previous results demonstrated that hyperglycemia can either directly (Figure 3A) or indirectly (*via* AGE-RAGE association) (Figure 3C) activate ROS. Therefore we next wanted to investigate if hyperglycemia mediated up-regulation of RAGE and binding of AGE to RAGE would also induce autophagy that results in subsequent NETs formation (as shown in Figure 2C). Incubation of PMNs with AGE in the presence and absence of antibody to RAGE for 30 and 120 showed an increased expression of LCIIIB after addition of AGE, however in the presence of anti-RAGE LCIIIB expression was markedly decreased. Bar graph showing densitometry measurements of LCIIIB/GAPDH expression (Figure 7C). To determine if this binding of AGE-RAGE is resulting in ROS generation, particularly hydrogen peroxide (H_2_0_2_), which further contributes to up-regulation of autophagy, we used catalase enzyme (150 units) to specifically block hydrogen peroxide (H_2_0_2_), and measured the expression of LCIIIB. Our results showed that after using catalase enzyme, the expression of LCIIIB is decreased confirming the role of hydrogen peroxide (H_2_0_2_) in the induction of autophagy (Figure 7D). Next, we wanted to correlate the association of AGE with RAGE interaction, subsequent ROS production and activation of autophagy on induction of NETosis. For that, we scavenged hydrogen peroxide (H_2_0_2_), using catalase (150 units) and checked for the expression of NETs using NETs generation assay and western blotting. Our results indicated that AGE resulted in up-regulation of autophagy which led to the formation of NETs. However, the addition of catalase (Figures 7E,F) lowered autophagy and NETosis, suggesting the role of AGE-mediated ROS, particularly hydrogen peroxide, in the induction of autophagy that led to NETosis. (Figure 7G).

### 3.9. Inhibition of autophagy prevents hyperglycemia-induced NETosis and decreases phagocytosis

**Figure 8:**
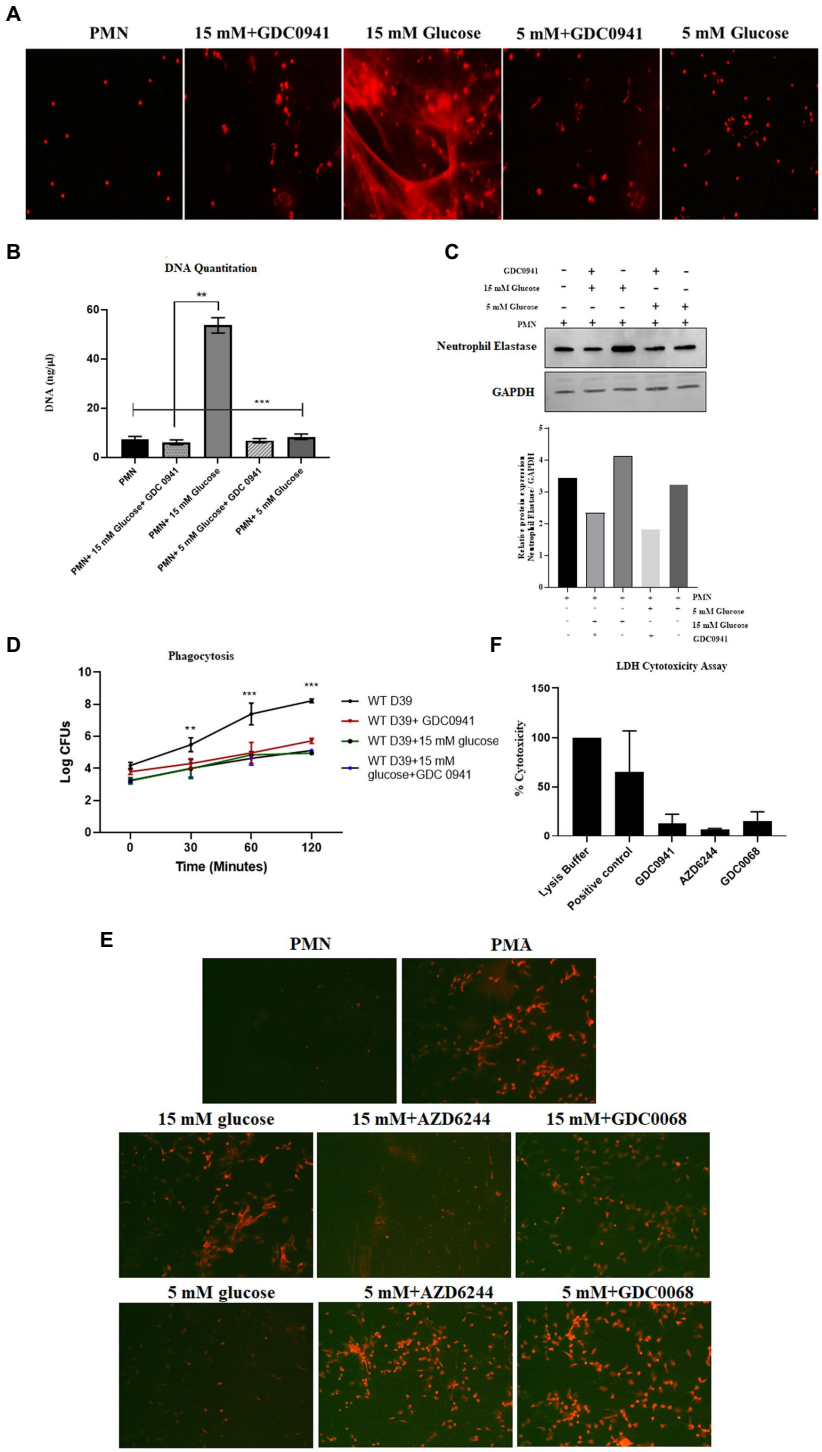
Inhibition of autophagy prevents hyperglycemia induced NETosis and decreases phagocytosis. **(A)** Fluorescent microscopy to visualize NETs in untreated (basal) PMNs as negative control, treated with 5 mM and 15 mM glucose in the presence and absence of GDC0941 (2 μM). **(B)** Bar graph showing DNA quantitation data. DNA (ng/μl) released after treating healthy PMNs with 5 mM and 15 mM glucose in the presence and absence of GDC0941, an inhibitor of autophagy. Data analyzed using one way ANOVA with multiple comparison test. Columns show mean; error bars are SEM. Statistical significance determined at *p* < 0.01. **(C)** Western blot analysis showing expression of neutrophil elastase protein in human PMNs left unstimulated, treated with normal glucose concentration (5 mM) and hyperglycemic glucose concentration (15 mM) in the presence and absence of an inhibitor of autophagy GDC0941. Lower panel showing GAPDH as a loading control. Bar graph showing densitometry analysis of neutrophil elastase/GAPDH of representative experiment. **(D)** Graph showing phagocytosis of D39 strain of *S. pneumoniae* by human PMNs in the presence and absence of autophagy inhibitor GDC0941 (2 μM). Data representative of three independent experiments analyzed using two way ANOVA. **(E)** Fluorescent microscopic images representing NETosis using propidium iodide DNA staining. PMNs isolated from healthy controls were treated with ERK inhibitor AZD6244 (10 μM), and pan-Akt inhibitor GDC0068 (2 μM) in the presence and absence of glucose (5 mM and 15 mM) to induce NETosis. PMA used as a positive control. The morphology of NETs observed after 4 h. Magnification: 20×. **(F)** LDH Cytotoxicity assay to determine cellular cytotoxicity upon incubating healthy PMNs with different inhibitors of autophagy AZD6244 (10 μM), GDC0068 (2 μM) and GDC0941 (2 μM). Graph shows the used concentrations of inhibitors are not cytotoxic for healthy PMNs.

Previous studies have shown that both autophagy and ROS are required for the formation of NETs (51). However, a three-way relationship between ROS, autophagy and NETosis remains to be determined. Additionally, it is also not known how conditions such as diabetes that impact both ROS and autophagy would modulate NETosis. We, therefore, investigated the hypothesis that hyperglycemia-mediated ROS activates autophagy which primes neutrophils for NETosis. To validate this hypothesis, we measured NETosis in PMNs in the presence and absence of inhibitors of autophagy. In individuals without diabetes, PMNs were stimulated with different concentrations of glucose (5 mM and 15 mM). The presence of PI-3kinase inhibitor (GDC0941; 2 μM) inhibited autophagy and blocked NETs formation, as indicated by qualitative analysis of NETs induction (Figure 8A) and quantitated by DNA quantitation analysis (Figure 8B) and measurement of levels of neutrophils elastase by western blotting and densitometry analysis (Figure 8C). Blocking autophagy also impacts phagocytosis as we observed a significant decrease in the log CFUs after incubating PMNs with PI-3Kinase inhibitor GDC0941, both in the presence and absence of 15 mM glucose. (Figure 8D). The PI3K–AKT–mTOR axis has widely been reported to connect autophagy and NETs formation, where inhibition of mTOR leads to enhances NET generation through activation of autophagy (52). Thus, to further validate the role of autophagy in induction of NETosis, we set out to block MAPK/Erk 1/2 and PI3K-I/AKT signaling pathways upstream of mTOR; a negative regulator of autophagy. We aimed to suppress mTOR expression using two inhibitors AZD6244 (inhibits upstream kinase of ERK), and GDC0068 a highly selective pan-Akt inhibitor, targeting Akt1/2/3. PMNs isolated from healthy individuals were treated with GDC0068 (2 μM), and AZD6244 (10 μM) in the presence and absence of 5 mM and 15 Mm glucose. Our results have demonstrated that the pan-Akt inhibitor GDC0068 and ERK inhibitor AZD6244 achieves significant upregulation in autophagy that results in increased NETosis in the presence of normoglycemic glucose concentration (i.e., 5 mM glucose). Thus confirming that activation of autophagy is required for induction of NETosis (Figure 8E). Lesser degree of NETosis observed with hyperglycemic glucose concentration (i.e., 15 mM glucose, may indicate that hyperglycemia triggers NETosis independently of mTOR). This however, needs further investigation. LDH cytotoxicity assay was performed to confirm that the concentrations of inhibitors AZD6244 (10 μM), GDC0941 (2 μM), GDC0068 (2 μM), used during the assays are not toxic to healthy PMNs. The percent cytotoxicity displayed by the three inhibitors is significantly lower as compared to the positive control. (Figure 8F).

## 4. Discussion

Hyperglycemia, an important downstream complication of T2D, has been implicated in the dysfunctioning of immune cells such as neutrophils (PMNs). Recent reports suggest that hyperglycemia leads to metabolic reprograming, characterized by excessive glycolysis and pentose phosphate pathway (PPP), and subsequent elevated levels of metabolites which feeds into neutrophil functions such as activation of NADPH-oxidase and production of ROS (53). This metabolic reprogramming has also been implicated in trained immunity, where both neutrophils and macrophages demonstrate a legacy of hyperglycemia, by epigenetic changes, leading to priming of macrophages and neutrophils into a more pro-inflammatory phenotype (54). Moreover, elevated levels of glucose and its associated metabolic reprograming in diabetes demonstrates marked increase in glycation of proteins and fatty acids through polyol and hexosamine pathways. End products thus made are referred to AGE (42, 55). Association of AGE to their cognate receptors on surface of PMNs leads to activation of protein kinase C (56, 57). These metabolic and biochemical disturbances, both inside and outside of PMNs, translates into increase in superoxide production, and activation of pro-inflammatory conditions in PMNs (53, 54, 58). While several reports present data on impact of hyperglycemic condition on impairments in PMNs functions, in particular induction of NETosis, none of these reports provide a mechanism for induction of NETosis in diabetes. We developed this study to address this gap in knowledge and performed a series of experiments to explain impairment in processes of NETosis and imbalance between induction of NETs and phagocytosis. Our results demonstrated spontaneous NETosis in conditions of hyperglycemia and in the presence of AGE. Moreover, PMNs isolated from those with poorly controlled T2D showed similar phenotype, where spontaneous NETosis was observed, in the absence of PMA. These results correlated with previous findings presented from several laboratories (30, 31) implicating hyperglycemia in spontaneous NETosis.

Binding of AGE with RAGE increase RAGE protein expression in “feed-forward” loop, which results in activation of cytosolic NADPH-oxidase and production of ROS (59, 60). Incubation of glycated-albumin demonstrated elevated surface expression of RAGE in healthy PMNs, whereas, in PMNs isolated from those with diabetes showed surface expression of RAGE which correlated with glycemic control. Interaction of AGE-RAGE showed increase levels of ROS and subsequent NETosis. High concentration of glucose has been reported to promote ROS mediated NFKB activation that further increase in the expression of RAGE receptor (61). These results further confirmed that hyperglycemic conditions and its associated metabolic impairments can sensitize/primes PMNs to NETs expression, while reducing phagocytosis. The low phagocytic capacity of PMNs in the presence of hyperglycemia and low bacterial-killing by diabetic PMNs, could be explained by the fact that spontaneous NETosis was reducing the number of available PMNs required for phagocytosis. Towards that we demonstrated a low level of pneumococcal phagocytosis as compared to NETosis in PMNs cultured in the conditions of hyperglycemia, or in PMNs isolated from those with diabetes. However, blocking NETosis by blocking ROS, restored phagocytosis, suggesting a ROS mediated mechanism that favors NETosis. Another reason for the observed decrease in phagocytosis could be the release of macrovesicles from dying PMNs which are priming neighboring PMNs to commit to NETosis. Alternatively, it was also likely that hyperglcyemia was inducing a downstream pathway that facilitated NETosis in comparison to phagocytosis. The observation that hyperglycemia increases NETosis while reducing phagocytosis was interesting and warranted further investigation.

Previous reports (62–64) have demonstrated glucose mediated autophagy in neurons and renal cells. Autophagy is a key quality control mechanism known to maintain cellular integrity (65, 66). In condition of hyperglycemia, mitochondrial depolarization, endoplasmic reticulum stress and miss folding of proteins leads to induction of autophagy, targeted to removal of miss folded proteins (67). These observations suggests that autophagy induction may be a default mechanism to prevent high glucose-induced cellular damage. Elaborating on the role of hyperglycemia in activation of autophagy and subsequent NETosis, we demonstrated that high glucose induces autophagy in human neutrophil in classical PI-3 K dependent fashion. The fact that we observed LCIIIB associated with PMNs isolated from those with poorly controlled diabetes, suggested that metabolic reprograming in response to hyperglycemia leads to induction of autophagy in diabetes. Autophagy leads to chromatin decondensation which is essential for NETosis (20). Therefore to confirm if autophagy in hyperglycemia will also lead to NETosis, we blocked formation of LCIIIB, using GDC0941, a PI-3kinase inhibitor, which inhibited autophagy and downstream NETosis, even in condition of hyperglycemia. Furthermore, we also observed a strong association of autophagy with NADPH-oxidase, suggesting that reactive oxygen species generated during hyperglycemia, conditions neutrophils for NETosis by activating autophagy. These observations are also supported by previous studies demonstrating that AGE binding to RAGE enhances the expression of Beclin and LCIIIB in cardiomyocytes, increased the number of autophagic vacuoles, and most importantly reduced cell viability in dose dependent manner. Inhibition of RAGE by pretreatment of PMNs with soluble RAGE, decreased Beclin-1 and LCIII expression (68–70). We made similar observations where inhibition of either NADPH oxidase or blocking of pathway upstream of autophagy attenuated NETosis.

In conclusion, our results provide insight into impairment in mechanisms of bacterial killing by neutrophils in the presence of hyperglycemia. Our study is the first to demonstrate that hyperglycemia directly and *via* a secondary mechanism involving AGE-RAGE association induces oxidative stress, through activation of NADPH-oxidase leading to activation of autophagy and spontaneous NETosis, while reducing phagocytosis. NETs thus generated were short-lived and disintegrated when incubated with pneumococci.

## 5. Conclusion

To our knowledge data presented in this paper provides a holistic understanding of how hyperglycemia induce spontaneous NETosis. We have demonstrated that hyperglycemia directly and *via* AGEs-RAGE interaction induces oxidative stress, at least in part through activation of NADPH oxidase, resulted in the activation of autophagy and ultimately lead to excessive NETosis and decreased phagocytosis in neutrophils. In conclusion, our findings provides a mechanism for the observed relationship between hyperglycemia and poor bactericidal activity. We were able to demonstrate that the link between hyperglycemia and poor bactericidal activity is elevated levels of ROS, which modulates autophagy switching the bactericidal activity from phagocytic killing to release of NETs.

## Data availability statement

The original contributions presented in the study are included in the article/Supplementary material, further inquiries can be directed to the corresponding author/s.

## Ethics statement

The studies involving human participants were reviewed and approved by Lahore University of Management Sciences (LUMS), and Shalamar Hospital Institutional Review Boards (IRB). The patients/participants provided their written informed consent to participate in this study.

## Author contributions

AnF and SM designed the research and wrote the manuscript. AnF, GH, SA, ZY, and KS performed the experiments. BY helped with the provision of blood samples. AmF critically reviewed the research design and the manuscript. All authors contributed to the article and approved the submitted version.

## Funding

This work is supported by the Higher Education Commission of Pakistan, grant 5931/Punjab/NRPU/HEC and grant 9319/Punjab/NRPU/HEC.

## Conflict of interest

The authors declare that the research was conducted in the absence of any commercial or financial relationships that could be construed as a potential conflict of interest.

## Publisher’s note

All claims expressed in this article are solely those of the authors and do not necessarily represent those of their affiliated organizations, or those of the publisher, the editors and the reviewers. Any product that may be evaluated in this article, or claim that may be made by its manufacturer, is not guaranteed or endorsed by the publisher.
