# Supplementary material for: Spontaneous NETosis in diabetes: A role of hyperglycemia mediated ROS and autophagy
Source: Front Med (Lausanne). 2023 Feb 20;10:1076690. doi: 10.3389/fmed.2023.1076690 (PMC9988915; doi:10.3389/fmed.2023.1076690)

Figure 1D and 5G

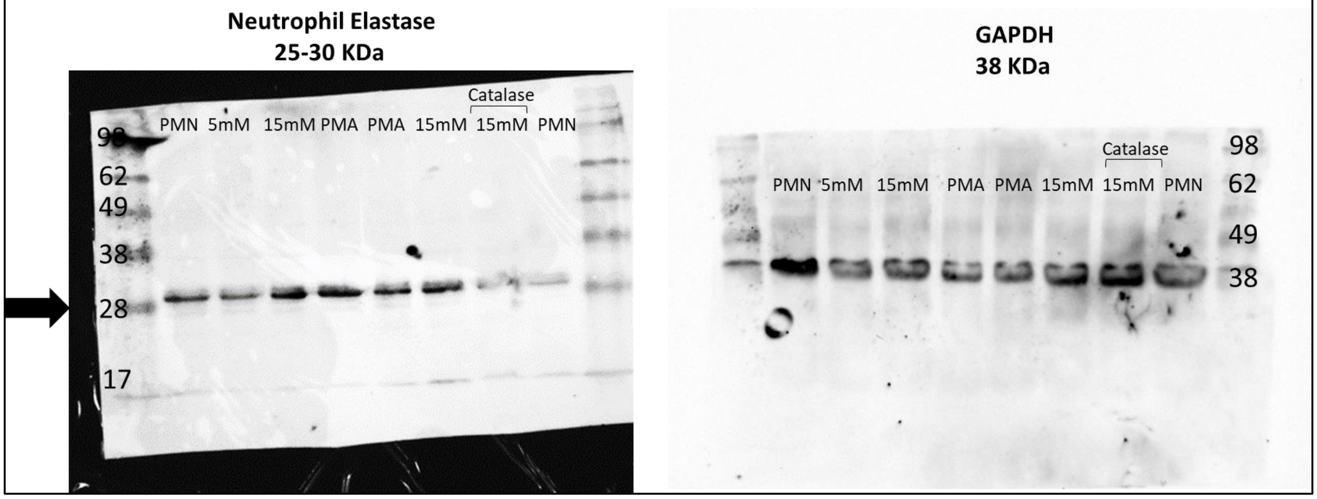

Figure 1I, 2B and 7B

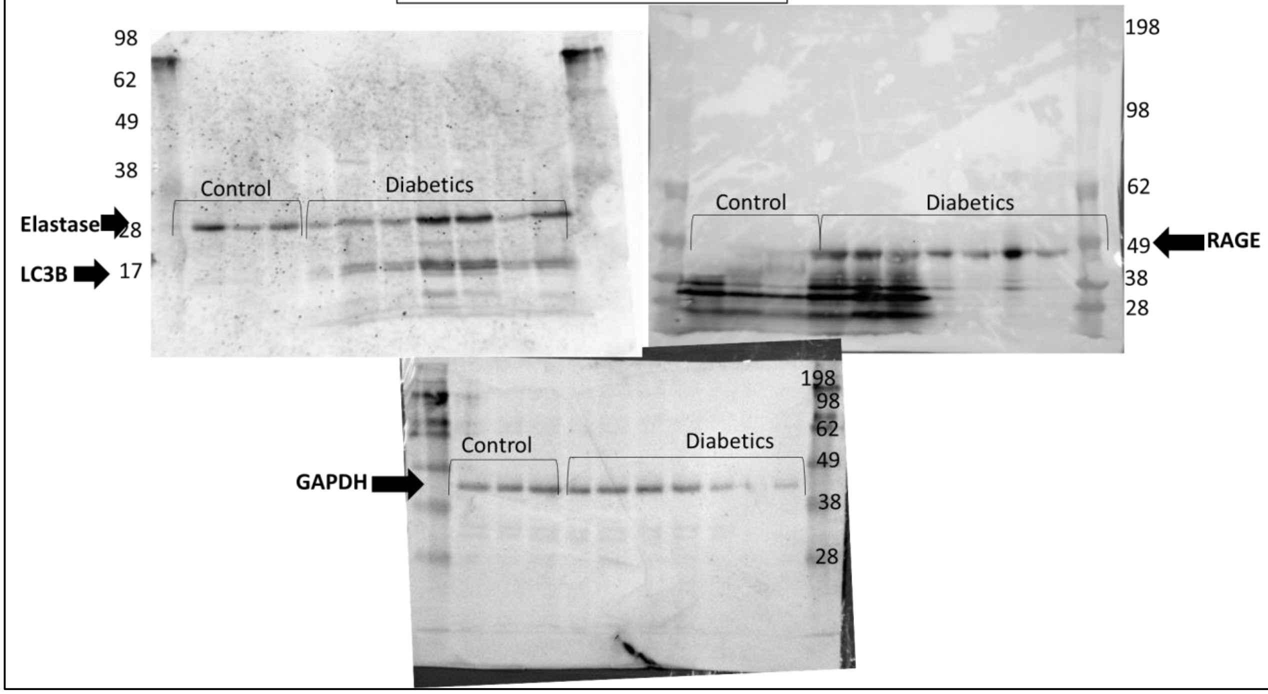

Figure 2A

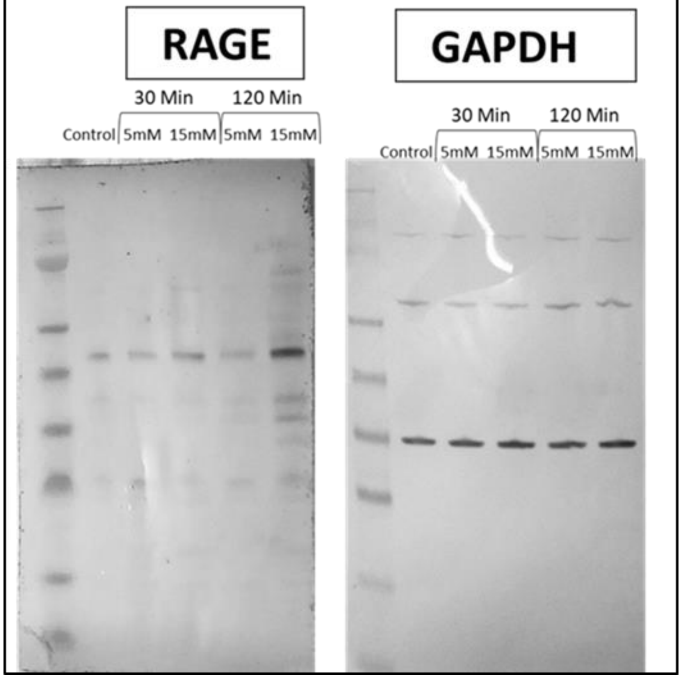

Figure 4E

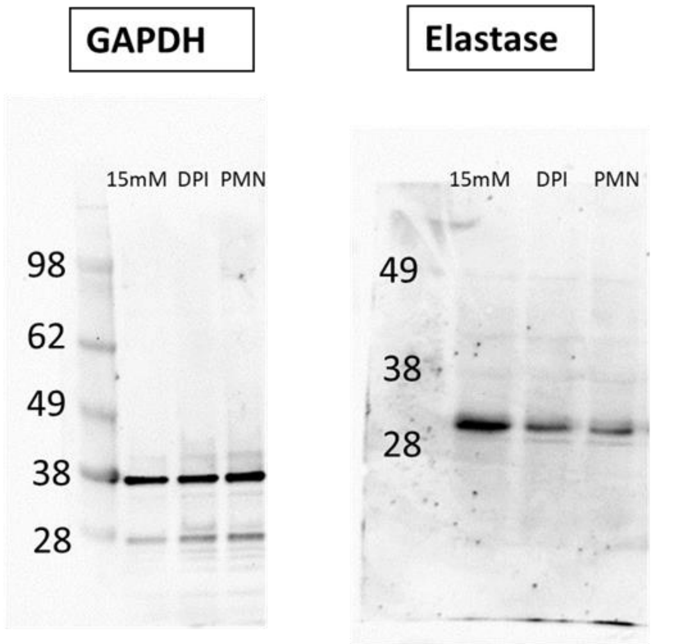

Figure 4A

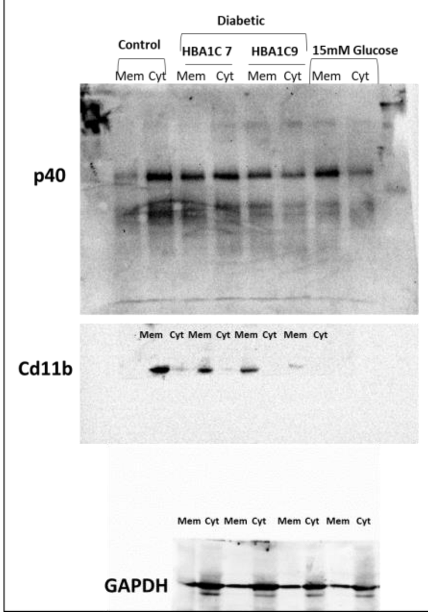

Figure 4B

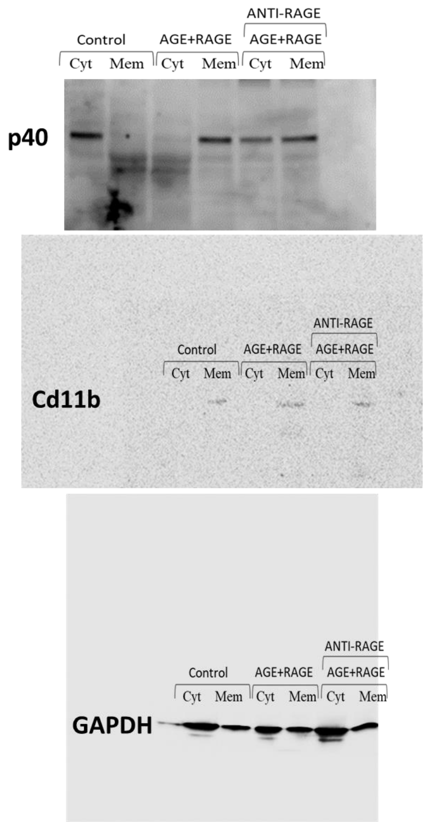

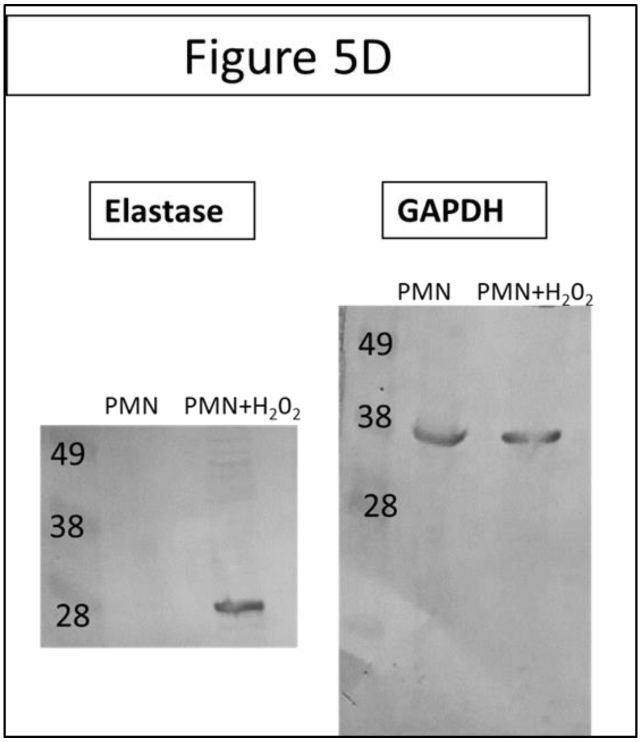

Figure 5D
Elastase
GAPDH
PMN
PMN+H₂0₂
49
38
28

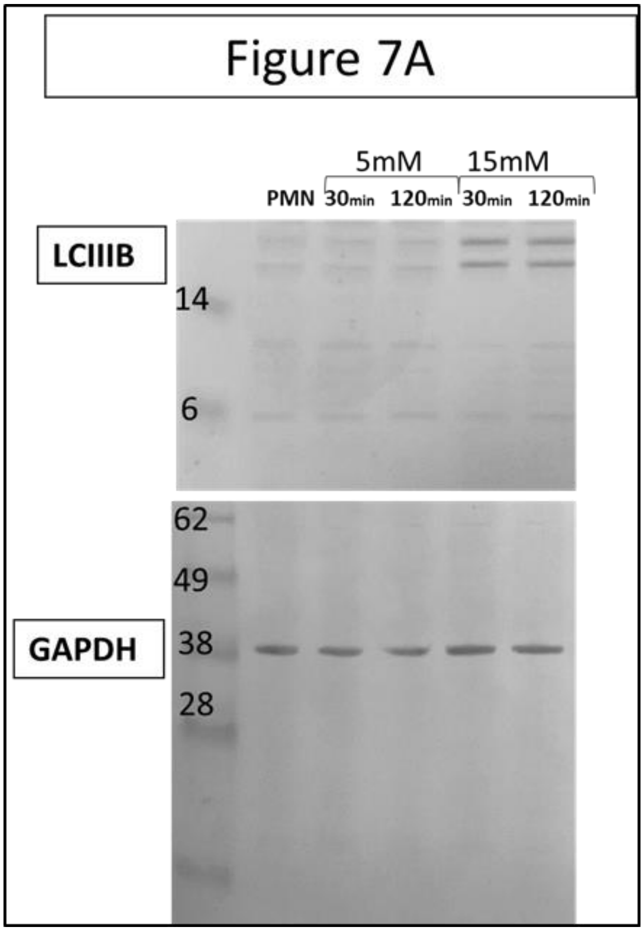

Figure 7A
5mM
15mM
PMN
30min
120min
30min
120min
LCIIIB
14
6
62
49
38
28
GAPDH

Figure 7B

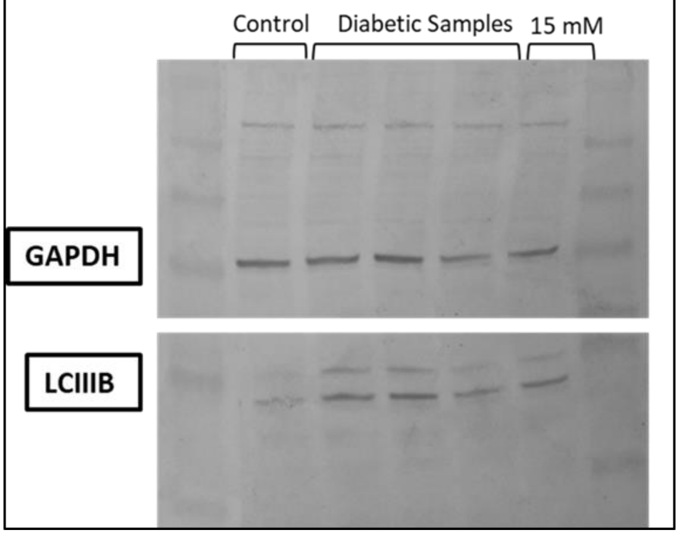

Figure 7C & 7D

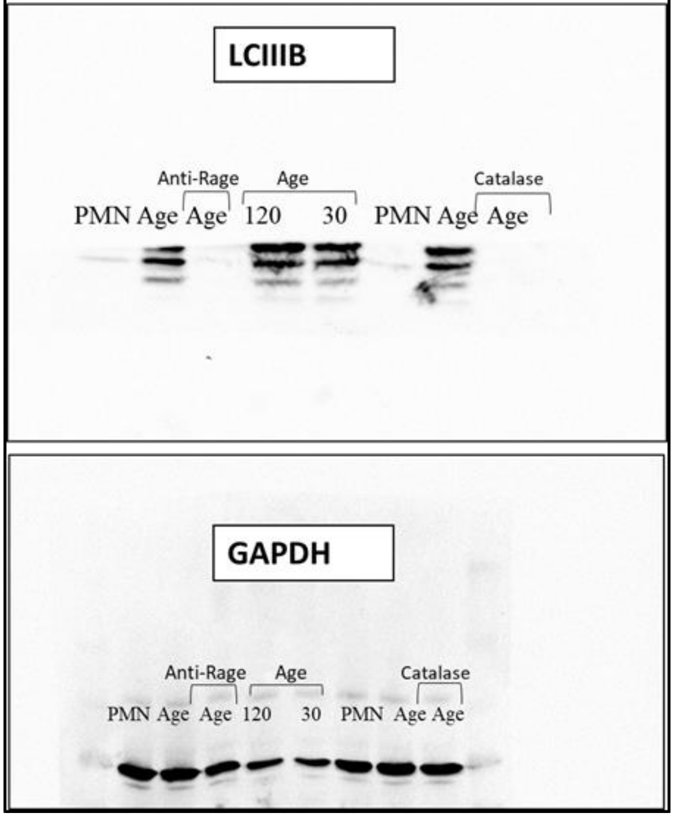

Figure 2E and 7G

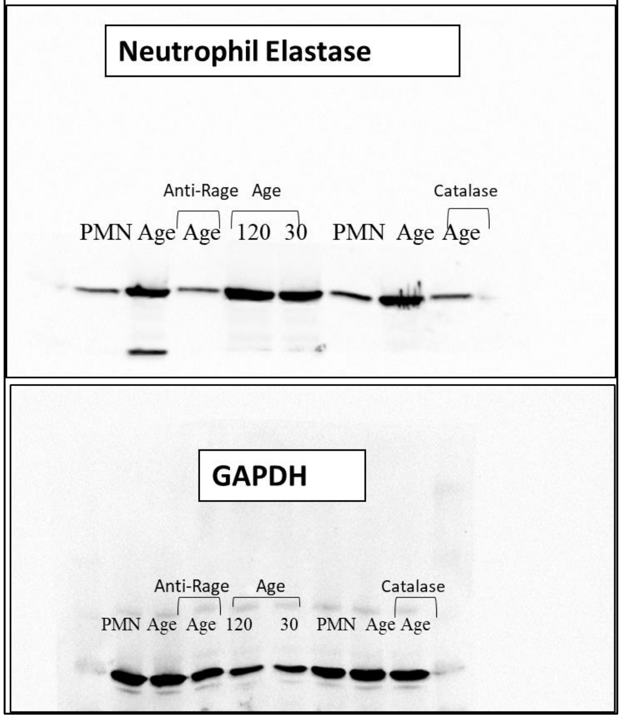

Figure 8C

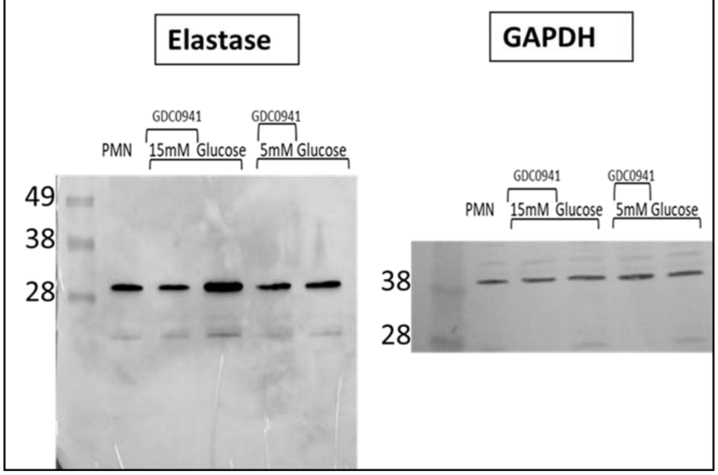

Supplement: Supplementary file 1 [file Data_Sheet_1.pdf]
